# Disability and costs of IHD attributable to the consumption of trans-fatty acids in Brazil

**DOI:** 10.1017/S1368980024001101

**Published:** 2024-05-10

**Authors:** Magda do Carmo Parajára, Aline Siqueira Fogal Vegi, Ísis Eloah Machado, Mariana Carvalho de Menezes, Eliseu Verly-Jr, Adriana Lúcia Meireles

**Affiliations:** 1 Programa de Pós-Graduação em Saúde e Nutrição, Escola de Nutrição, Universidade Federal de Ouro Preto, Ouro Preto, Minas Gerais, Brazil; 2 Departamento de Medicina de Família, Saúde Mental e Coletiva, Escola de Medicina, Universidade Federal de Ouro Preto, Ouro Preto, Minas Gerais, Brazil; 3 Departamento de Nutrição Clínica e Social, Escola de Nutrição, Universidade Federal de Ouro Preto, Ouro Preto, Minas Gerais, Brazil; 4 Departamento de Epidemiologia, Instituto de Medicina Social, Universidade do Estado do Rio de Janeiro, Rio de Janeiro, Rio de Janeiro, Brazil

**Keywords:** Global burden of disease, Noncommunicable diseases, Trans-fatty acids, Healthcare costs, Costs and cost analysis, Cost savings

## Abstract

**Objective:**

To estimate the disability and costs of the Brazilian Unified Health System for IHD attributable to trans-fatty acid (TFA) consumption in 2019.

**Design:**

This ecological study used secondary data from the Global Burden of Disease (GBD) Study 2019 to estimate the years lived with disability from IHD attributable to TFA in Brazil in 2019. Data on direct costs (purchasing power parity: 1 Int$ = R$ 2·280) were obtained from the Hospital and Ambulatory Information Systems of the Brazilian Unified Health System. Moreover, the total costs in each state were divided by the resident population in 2019 and multiplied by 10 000 inhabitants. The relationship between the socio-demographic index, disease and economic burden was investigated.

**Setting:**

Brazil and its twenty-seven states.

**Participants:**

Adults aged ≥ 25 years of both sexes.

**Results:**

IHD attributable to TFA consumption resulted in 11 165 years lived with disability (95 % uncertainty interval 932–18 462) in 2019 in Brazil. A total of Int$ 54 546 227 (95 % uncertainty interval 4 505 792–85 561 810) was spent in the Brazilian Unified Health System in 2019 due to IHD attributable to TFA, with the highest costs of hospitalisations, for males and individuals aged ≥ 50 years or over. The highest costs were observed in Sergipe (Int$ 6508/10 000; 95 % uncertainty interval 576–10 265), followed by the two states from the South. Overall, as the socio-demographic index increases, expenditures increase.

**Conclusions:**

TFA consumption results in a high disease and economic IHD burden in Brazil, reinforcing the need for more effective health policies, such as industrial TFA elimination, following the international agenda.

## Graphical abstract

**Figure:**
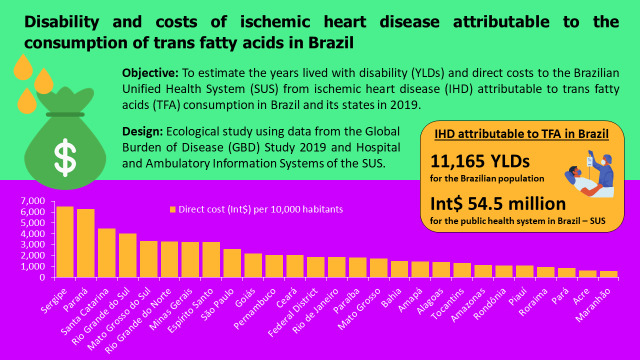

Over the last three decades, IHD has consistently been ranked as one of the leading causes of death and loss of health in Brazil^(1)^. Among noncommunicable diseases (NCD), CVD are recognised as a significant global public health issue because they cause premature mortality, have poor survival, impact health and quality of life, reduce workforce productivity, threaten economic prosperity due to enhanced healthcare costs and create enormous disparities in opportunity^(2,3)^. In the face of demographic, epidemiological and nutritional transition, there has been an increase in life expectancy and NCD (including CVD) and, consequently, in the impact on morbidity and disabilities caused by them. It is perceived that, in isolation, mortality measures would not be most adequate to describe the overall population health status since NCD often have non-fatal impacts on health; then, individuals live with the disease and its consequences for many years^(4)^. Also, the poorest and most vulnerable individuals regionally and at a subnational level are at the highest risk of developing CVD and are least likely to have access to detection and control, even with the rising healthcare expenditure caused by them^(2,3)^. The CVD burden in low- and middle-income countries is challenging^(3)^.

The huge CVD burden is attributable to environmental, metabolic and behavioural risk factors, including unhealthy diet^(3,5,6)^. For example, trans-fatty acids (TFA), a preventable dietary risk factor, have a good level of evidence as a critical risk factor for CVD^(7)^. TFA are defined as unsaturated fats that have at least one double bond in the transconfiguration and are divided into two groups: TFA naturally produced by ruminants, found in meat and dairy products, and industrial TFA (iTFA), found mainly in partially hydrogenated vegetable oils (PHO)^(8)^.

Given the alarming burden of CVD, the WHO has been implementing an action package called REPLACE, which aims to reduce and eliminate iTFA globally by 2023^(9)^. Following this global agenda, the Member States of the Pan American Health Organization (PAHO), which Brazil participates, approved the *Plan of Action for the Elimination of Industrially Produced Trans-Fatty Acids 2020–2025* ^(10)^. Consequently, in 2019, in Brazil, a resolution to limit TFA to 2 % of total fats in all foods between 1 July 2021 and 1 January 2023 and to ban the production and use of PHO from January 2023 was published^(11)^.

In Brazil, although a temporal trend of IHD attributable to TFA from 1990 to 2019 revealed a decrease of approximately 60 % in mortality and disability-adjusted life years’ age-standardised rates, this burden is still relevant to the country, and more effective policies, such as TFA ban, only implemented in 2021, could have more marked effects on this burden^(12)^. A modelling study showed that banning PHO could prevent or postpone approximately 10 500 deaths (95 % uncertainty interval (95 % UI) 9963−10 909) in the Brazilian population in 2018^(13)^.

A systematic analysis of the health and economic burden of TFA consumption may reinforce the importance of reducing iTFA levels in Brazil and provide benchmarks for policy and decision-makers^(3,9,13)^. Moreover, expenses arising from premature deaths and disabilities caused by NCD threaten the efficiency and sustainability of health systems^(14)^. To the best of our knowledge, the disability and cost of the disease attributable to TFA in the Brazilian Unified Health System (SUS) have not yet been investigated. Therefore, this study aimed to estimate the years lived with disability (YLD) and the direct costs to the SUS from IHD attributable to TFA consumption in Brazil and its states in 2019.

## Methods

### Study design, sources of data and population

This descriptive ecological study used secondary data from the Global Burden of Disease (GBD) Study 2019, publicly available at https://ghdx.healthdata.org/ and retrieved in March 2023, to measure disease burden. GBD 2019, led by the Institute for Health Metrics and Evaluation (IHME), aims to quantify health loss in populations worldwide, ensuring comparative, detailed and current results for evidence-based policymaking. Further details of the GBD data, methods and results have been previously reported^(1,6)^.

Economic burden estimation of the Brazilian public health system (SUS) was based on publicly available information from the Department of Informatics of the Unified Health System, Health Ministry, at https://datasus.saude.gov.br/, retrieved in May 2022. The Department of Informatics of the Unified Health System allows access to transparent information on procedures and service provider payments^(14)^. Specifically, the Outpatient Information System (SIA/SUS) and Hospital Information System (SIH/SUS) databases were used to estimate costs. Both systems provide values practised in the Brazilian public health system and those transferred to health institutions that carry out health actions and services for the SUS^(14)^.

The disease and economic burden of IHD attributable to TFA were estimated for the Brazilian population aged ≥ 25 years in 2019.

### Disease burden

Disability was estimated using YLD (obtained from GBD 2019), which are understood as the years of healthy life lost. YLD were calculated by multiplying the prevalence of a sequela by the disability weights of diseases and injuries for that sequela^(1)^. Exposure, the consumption of TFA, is defined in the GBD 2019 as any intake (in % daily energy) of TFA from all sources^(6)^. The GBD 2019 sourced the information about TFA intake from national sales data provided by Euromonitor Passport^(6)^. To split the data into standard age groups, GBD defined the global age and patterns of the dietary factor using data obtained from 24 h dietary recall. Afterwards, GBD utilised the recognised age patterns to divide the sales data into standard age categories^(6,15)^. For continuous data not originating from the 24 h dietary recall, considered the gold standard by the GBD, such as TFA consumption data, various adjustments are applied to render them more consistent and suitable for modelling^(6)^. Additionally, the spatiotemporal Gaussian process regression framework still allows other types of information that have plausible relationships with dietary intakes, such as country-level covariates, to control and adjust for data biases. However, for TFA, the GBD 2019 did not include any adjusting covariates in the model^(6)^.

The attributable burden for the risk-outcome pair is measured by GBD using a comparative risk assessment, also called the population attributable fraction (PAF). The PAF were obtained from GBD 2019 and corresponded to the proportion of YLD that could be avoided if the population achieved counterfactual exposure in the past (i.e. the theoretical minimum risk exposure level)^(6)^. For TFA, the theoretical minimum risk exposure level represents no TFA intake^(6)^. In addition to the theoretical minimum risk exposure level, the PAF includes two other inputs: the average daily TFA intake and the relative risks to the risk-outcome pair (see online supplementary material, Supplemental Tables S1 and S2). Then, the attributable YLD were calculated by GBD multiplying PAF for each age, sex, location and year by the outcome quantity, enabling stratified analyses^(6)^. The PAF of IHD attributable to TFA are presented in online supplementary material, Supplemental Supplementary Table S3.

GBD select the dietary risk factors based on some criteria: the importance of the risk factor to disease burden or policy; sufficient data to estimate risk factor exposure; the strength of the epidemiological evidence supporting a causal relationship between risk factor exposure and disease, along with the accessibility of data to measure the extent of this relationship for each unit of exposure change; and substantial evidence of the applicability of the effects to diverse populations^(15)^. The World Cancer Research Fund criteria were used by GBD for grading convincing or probable evidence of risk-outcome pairs^(6)^. Then, based on published systematic reviews, GBD identified IHD as the only outcome attributed to the TFA consumption^(6)^.

IHD is a disease of the coronary arteries, mainly from atherosclerosis, leading to myocardial infarction or ischaemia and stable angina^(1)^, mapped to the GBD 2019 using the International Classification of Diseases 10th Revision (ICD-10) codes: I20–I25·9, Z82·4–Z82·49^(16)^. Furthermore, GBD 2019 considers two metabolic mediators in the physiological pathway between TFA consumption and IHD: high LDL-c and systolic blood pressure (ICD-10 codes E78·0 and I10, respectively)^(6)^.

The number, crude and age-standardised rate of YLD from IHD attributable to TFA in Brazil and its twenty-seven federative units, referred to here as states and divided into five regions (see online supplementary material, Supplemental Fig. S1), in 2019 were described. The YLD attributable burden estimates in states were expressed in quartiles. GBD uses a standard GBD world population to calculate age-standardised rates. The rates were expressed per 100 000 inhabitants in this study.

### Economic burden

Direct costs in the public health system related to outpatient care and hospitalisations were evaluated in this study, including the following expenditures: specialised medical consultations, hospital admissions, medications administered in hospital outpatient settings, orthoses and prostheses, and complementary procedures for secondary and tertiary care. The expenses did not include primary healthcare or medications beyond the scope of secondary and tertiary healthcare services.

SIA/SUS and SIH/SUS identify the cost per procedure related to IHD using the ICD-10 codes^(14)^. These codes were used to link the costs and PAF obtained from GBD 2019 by sex, age, location and year^(17)^. The Individualized Outpatient Production Bulletin (BPA-I) and the Authorizations for High Complexity Procedures (APAC) were used to obtain data from SIA/SUS in 2019, 2020 and 2021, while the reduced Hospital Admission Authorization (AIH) was used for SIH/SUS for the same period. The years 2020 and 2021 were included to consider information from 2019, which was corrected in subsequent years. Subsequently, only the 2019 data were retained. In addition, to avoid age typographical errors, individuals aged > 110 years were excluded.

Cost data were extracted and processed using the R Microdatasus package^(18)^. The Stata ICD-10 package version 13 was used to group the procedures in SIA/SUS and SIH/SUS and the IHD attributable to TFA consumption from GBD 2019 through the ICD-10 codes. Subsequently, the IHD costs attributable to TFA consumption in 2019 were obtained by multiplying the total cost estimated for IHD by the respective PAF for each sex, age group and location. In addition, to remove the effect of population size, the total costs per state were divided by the estimated resident population in the respective state in 2019^(19)^ and multiplied by 10 000 inhabitants.

All costs were estimated in Brazilian reais (R$) and then converted into international dollars (Int$), a hypothetical unit of currency equivalent to the purchasing power of one US dollar (US$), considering the 2019 purchasing power parity (Int$ 1 = R$ 2·280)^(20)^.

### Disease and economic burden and their relationship with the socio-demographic index

The correlations of the age-standardised YLD rate and the economic burden of IHD attributable to TFA consumption with the socio-demographic index (SDI) in 2019 were assessed. SDI is a composite metric developed by the GBD related to health outcomes^(6)^. Briefly, SDI ranges from 0 (less developed) to 1 (most developed) and comprises income per capita, mean years of schooling for those aged ≥ 15 years and fertility rate in females under 25 years^(1,6)^.

### Statistical analysis

The estimates were presented for both sexes, all ages and the country pooled and stratified by type of healthcare (outpatient care and hospitalisation), sex, age group and state, with 95 % UI. The 95 % UI consist of a range of values probable to include the correct estimate of health loss for a specific cause. They are calculated by GBD 2019 to incorporate the uncertainty of parameters (such as the exposure, relative risk, ideal level of intake and mortality) through Monte Carlo simulation iterations, wherein the UI are the 2·5th and 97·5th values of the ordered 1000 values and are chosen after repeating all calculations 1000 times using one draw of each parameter^(15)^. These percentiles represent the lower and upper bounds of the interval, respectively.

QGIS version 3.22.3 was used to create maps of the disease burden. Additionally, costs were analysed using STATA version 13.0.

## Results

In 2019, IHD attributable to TFA consumption caused 11 165 YLD (95 % UI 932–18 462) in the Brazilian population. The crude YLD rate was 5·15/100 000 (95 % UI 0·43–8·52). The results are shown in online supplementary material, Supplemental Table S4.

**Fig. 1:**
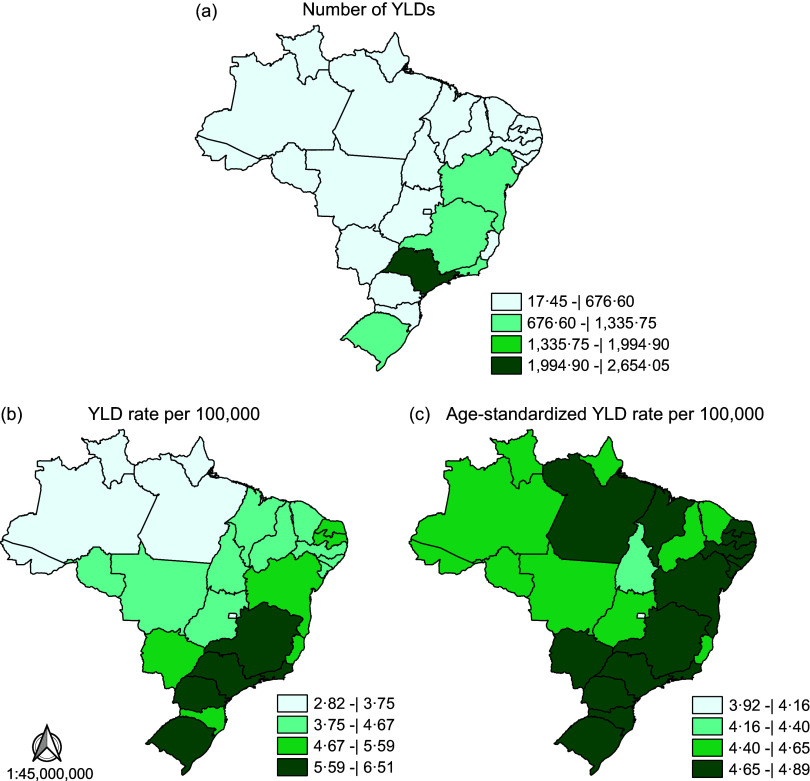
Number and rates of years lived with disability, per 100 000, for IHD attributable to trans-fatty acid consumption in Brazil, 2019. YLD: years lived with disability

Figure 1 (see online supplementary material, Supplemental Table S4) shows the total number, crude rates and age-standardised rates of YLD from IHD attributable to TFA consumption for the twenty-seven Brazilian states. In general, the highest YLD number was in states from the Northeast, South and Southeast states, especially in São Paulo, the state with the highest value: 2654·05 YLD (95 % UI 221·07–4399·80) (Fig. 1(a)). The crude rates revealed almost two more YLD in South and Southeast states when compared with those from the North (Fig. 1(b)). Although the age-standardised rates were quite similar across the country, the Federal District, in the Central-West region, presented the lowest rates of YLD: 3·92/100 000 (95 % UI 0·33–6·44) (Fig. 1(c)).

The resulting total direct cost to the SUS in Brazil in 2019 with IHD attributable to TFA consumption was Int$ 54 546 227 (95 % UI 4 505 792–85 561 810), as shown in Table 1. The higher cost share (93·72 %) was for the hospitalisations (Int$ 51 121 821; 95 % UI 4 195 620–80 168 034), whereas the costs of outpatient care were estimated at Int$ 3 430 406 (95 % UI 310 172–5 393 776). Comparing the costs by sex, male individuals had higher costs (Int$ 35 006 662; 95 % UI 2 708 429–55 099 866) than females (Int$ 19 539 565; 95 % UI 1 797 363–30 461 944), even when compared by age group and type of healthcare. As expected, there was a general trend of cost increase with age, with the highest expenditure concentrated in individuals aged ≥ 50 years.

**Table 1:** The direct cost of IHD attributable to the trans-fatty acid consumption to the Unified Health System in Brazil by type of procedure, sex and age group, 2019

		SIA		SIH		Total	
Age group (years)	Sex	Int$	95 % UI	Int$	95 % UI	Int$	95 % UI
25–29	Male	1681	107–2629	106 957	7072–166 688	108 638	7179–169 317
	Female	1408	102–2215	45 603	3277–70 721	47 011	3379–72 936
	Both	3089	209–4844	152 560	10 349–237 409	155 649	10 558–242 253
30–34	Male	1935	128–3104	249 725	16 253–399 280	251 660	16 381–402 384
	Female	1817	139–2834	113 808	8251–177 642	115 625	8390–180 476
	Both	3752	267–5938	363 533	24 504–576 922	367 285	24 771–582 860
35–39	Male	3693	235–5818	654 847	42 309–1 026 718	658 540	42 544–1 032 536
	Female	2976	232–4647	278 141	21 925–429 445	281 117	22 157–434 092
	Both	6669	467–10 465	932 988	64 234–1 456 163	939 657	64 701–1 466 628
40–44	Male	5363	371–8506	1 243 121	85 953–1 963 679	1 248 484	86 324–1 972 185
	Female	3829	298–6045	611 640	47 309–953 233	615 469	47 607–959 278
	Both	9192	669–14 551	1 854 761	133 262–2 916 912	1 863 953	133 931–2 931 463
45–49	Male	8306	570–13 218	2 552 043	171 300–4 027 756	2 560 349	171 870–4 040 974
	Female	6387	544–9898	1 218 778	103 794–1 872 099	1 219 165	104 338–1 881 997
	Both	14 693	1114–23 116	3 770 821	275 094–5 899 855	3 779 514	276 208–5 922 971
50–54	Male	15 518	1081–24 821	4 292 398	295 557–6 769 326	4 307 916	296 638–6 794 147
	Female	12 518	1030–19 624	1 919 303	158 943–2 986 870	1 931 821	159 973–3 006 494
	Both	28 036	2111–44 445	6 211 701	454 500–9 756 196	6 239 737	456 611–9 800 641
55–59	Male	32 988	2449–52 546	5 581 717	417 261–8 801 608	5 614 705	419 710–8 854 154
	Female	29 057	2588–46 058	2 621 758	235 613–4 140 142	2 650 815	238 201–4 186 200
	Both	62 045	5037–98 604	8 203 475	652 874–12 941 750	8 265 520	657 911–13 040 354
60–64	Male	63 644	4895–100 637	6 051 446	475 073–9 469 378	6 115 090	479 968–9 570 015
	Female	62 337	5726–97 715	3 002 874	274 796–4 671 548	3 065 211	280 522–4 769 263
	Both	125 981	10 621–198 352	9 054 320	749 869–14 140 926	9 180 301	760 490–14 339 278
65–69	Male	114 065	9327–179 963	5 138 951	437 843–8 119 338	5 253 016	447 170–8 299 301
	Female	114 745	10 622–179 849	2 894 115	271 233–4 520 254	3 008 860	281 855–4 700 103
	Both	228 810	19 949–359 812	8 033 066	709 076–12 639 592	8 261 876	729 025–12 999 404
70–74	Male	188 959	15 772–298 638	3 632 432	294 388–5 701 393	3 821 391	310 160–6 000 031
	Female	183 065	18 060–285 645	2 299 603	227 580–3 597 230	2 482 668	245 640–3 882 875
	Both	372 024	33 832–584 283	5 932 035	521 968–9 298 623	6 304 059	555 800–9 882 906
75–79	Male	255 868	21 639–400 325	2 213 135	184 971–3 451 561	2 469 003	206 610–3 851 886
	Female	233 892	23 205–363 804	1 575 579	154 471–2 435 812	1 809 471	177 676–2 799 616
	Both	489 760	44 844–764 129	3 788 714	339 442–5 887 373	4 278 474	384 286–6 651 502
80–84	Male	384 848	32 977–614 853	1 045 347	89 863–1 648 643	1 430 195	122 840–2 263 496
	Female	349 888	36 108–544 269	837 058	86 011–1 295 943	1 186 946	122 119–1 840 212
	Both	734 736	69 085–1 159 122	1 882 405	175 874–2 944 586	2 617 141	244 959–4 103 708
85–89	Male	317 984	27 068–506 128	382 434	33 212–603 473	700 418	60 280–1 109 601
	Female	296 830	27 266–462 947	349 324	32 186–540 692	646 154	59 452–1 003 639
	Both	614 814	54 334–969 075	731 758	65 398–1 144 165	1 346 572	119 732–2 113 240
90–94	Male	231 142	20 266–364 774	81 001	7147–127 384	312 143	27 413–492 158
	Female	221 348	21 598–344 163	99 461	9323–154 565	320 809	30 921–498 728
	Both	452 490	41 864–708 937	180 462	16 470–281 949	632 952	58 334–990 886
+95	Male	143 281	12 341–228 931	11 833	1001–18 750	155 114	13 342–247 681
	Female	141 034	13 428–219 172	17 389	1705–26 863	158 423	15 133–246 035
	Both	284 315	25 769–448 103	29 222	2706–45 613	313 537	28 475–493 716
**Total**	Male	1 769 275	149 226–2 804 891	33 237 387	2 559 203–52 294 975	35 006 662	2 708 429–55 099 866
Int$ (95 % UI)	Female	1 661 131	160 946–2 588 885	17 884 434	1 636 417–27 873 059	19 539 565	1 797 363–30 461 944
	Both	3 430 406	310 172–5 393 776	51 121 821	4 195 620–80 168 034	54 546 227	4 505 792–85 561 810

95 % UI: 95 % uncertainty interval; Int$: International dollar; SIA: Outpatient Information System; SIH: Hospital Information System.

Including direct costs related to the mediators between TFA consumption and IHD, LDL-c and systolic blood pressure would increase by more than Int$ 2 033 644 (95 % UI 165 656–3 176 781), totalling almost Int$ 57 million in expenditure on the Brazilian health system (see online supplementary material, Supplemental Table S5).

**Fig. 2:**
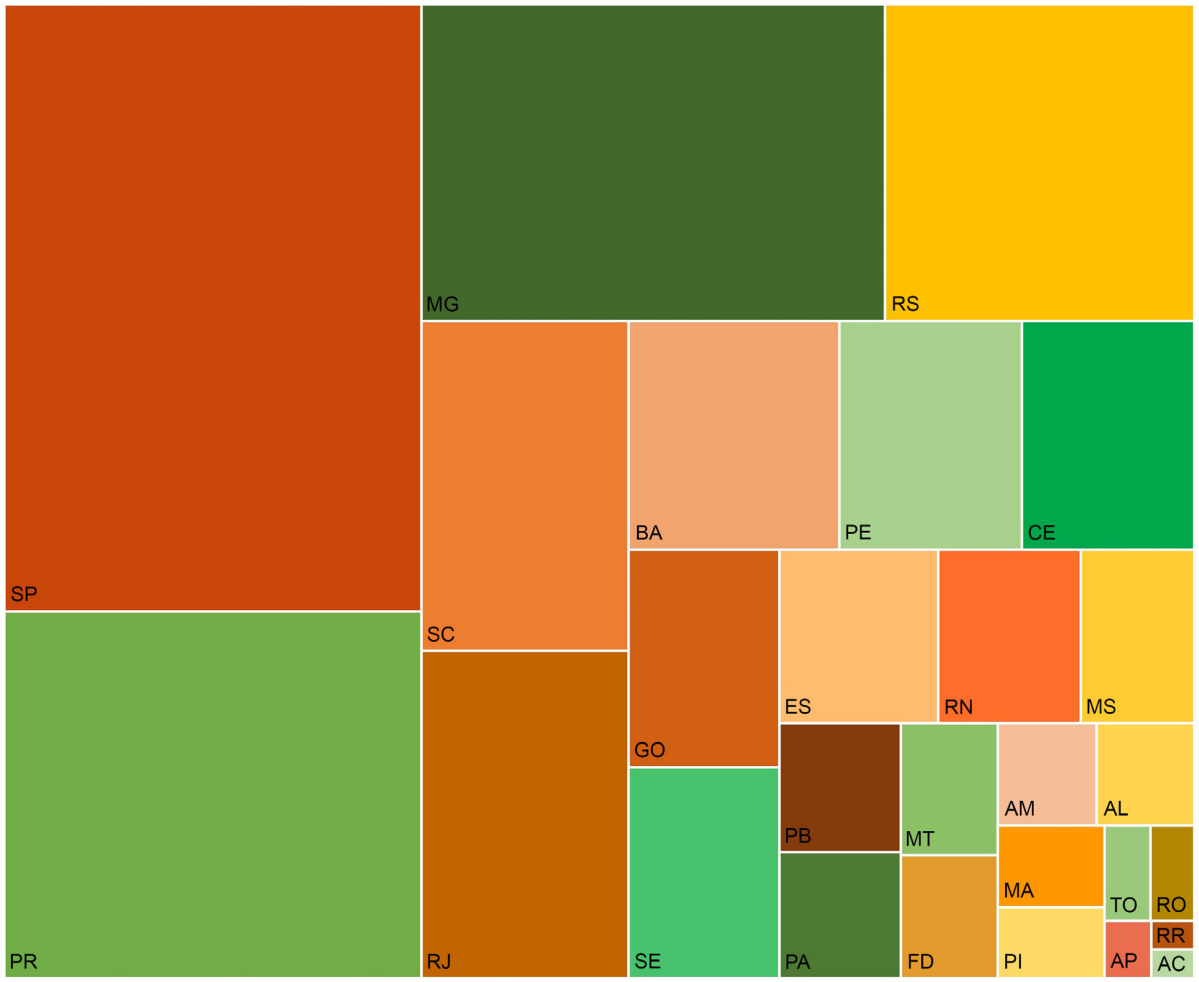
The direct cost (Int$) of IHD attributable to the trans-fatty acid consumption to the Unified Health System in Brazil by states, 2019. AC: Acre; AP: Amapá; AM: Amazonas; PA: Pará; RO: Rondônia; RR: Roraima; TO: Tocantins; AL: Alagoas; BA: Bahia; CE: Ceará; MA: Maranhão; PB: Paraíba; PE: Pernambuco; PI: Piauí; RN: Rio Grande do Norte; SE: Sergipe; FD: Federal District; GO: Goiás; MT: Mato Grosso; MS: Mato Grosso do Sul; ES: Espírito Santo; MG: Minas Gerais; RJ: Rio de Janeiro; SP: São Paulo; PR: Paraná; RS: Rio Grande do Sul; SC: Santa Catarina; Int$: international dollar

As shown in Fig. 2 (see online supplementary material, Supplemental Table S6), from the top ten higher expenditures with IHD attributable to TFA to the SUS at the state level in 2019, the first six states were from the South and Southeast. São Paulo, Paraná and Minas Gerais had the highest costs. The states from the North, such as Acre, Roraima and Amapá, spent less.

**Fig. 3:**
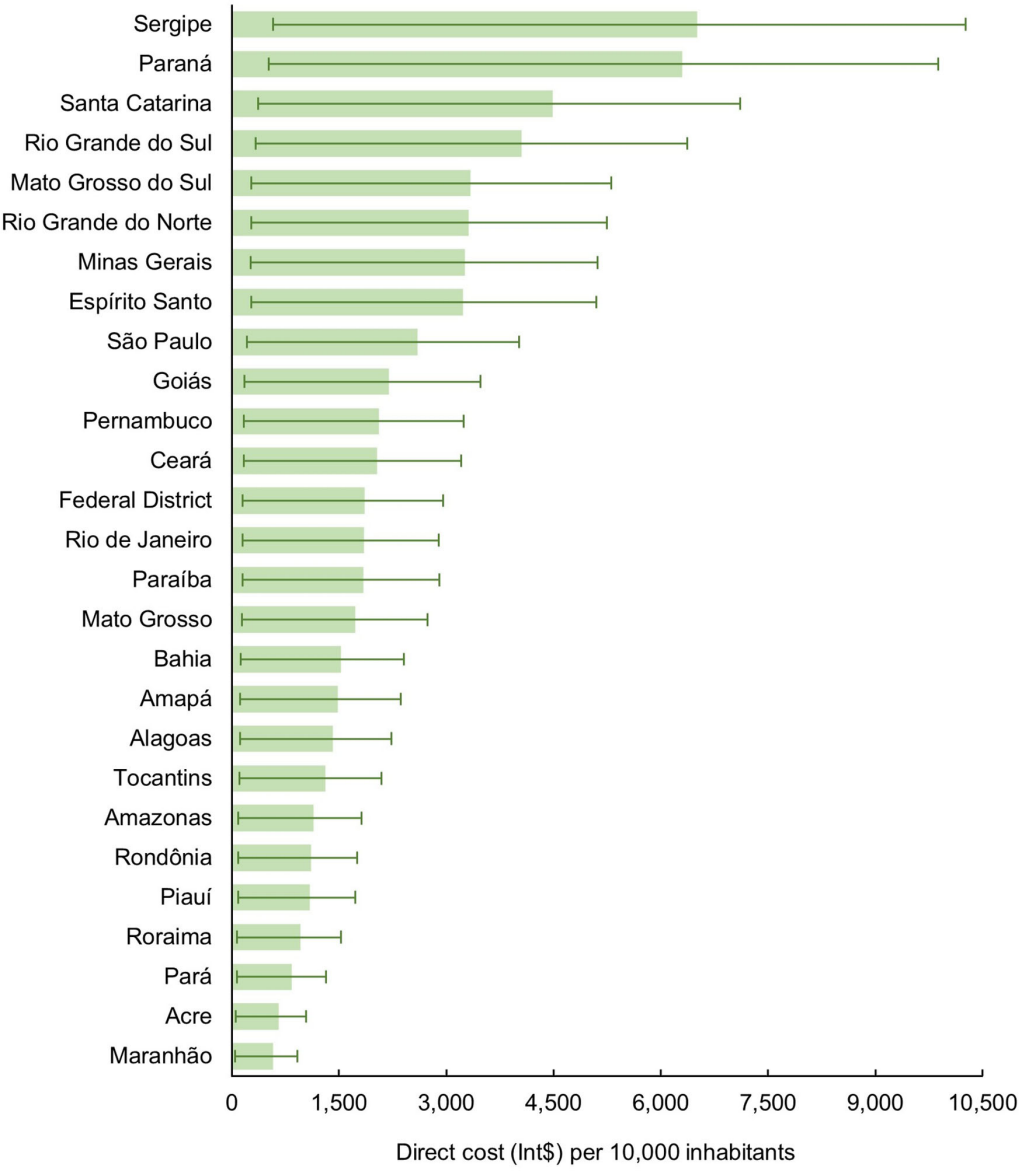
The direct cost (Int$) per 10 000 inhabitants of IHD attributable to the trans-fatty acid consumption to the Unified Health System in Brazil by states, 2019. Int$: international dollar

Additionally, to better understand and compare the states that contributed the highest cost to the SUS, we considered the size of their resident population and these costs for every 10 000 inhabitants (Fig. 3, see online supplementary material, Supplemental Table S7). The three highest costs were observed in Sergipe (Int$ 6508/10 000; 95 % UI 576–10 265), from the Northeast region, followed by Paraná (Int$ 6296/10 000; 95 % UI 520–9883) and Santa Catarina (Int$ 4490/10 000; 95 % UI 369–7111), both from the South. On the contrary, Maranhão (Int$ 581/10 000; 95 % UI 48–920), from the Northeast region, followed by Acre (Int$ 655/10 000; 95 % UI 51–1038) and Pará (Int$ 840/10 000; 95 % UI 71–1322), both from the North, spent less with IHD attributable to TFA.

**Fig. 4:**
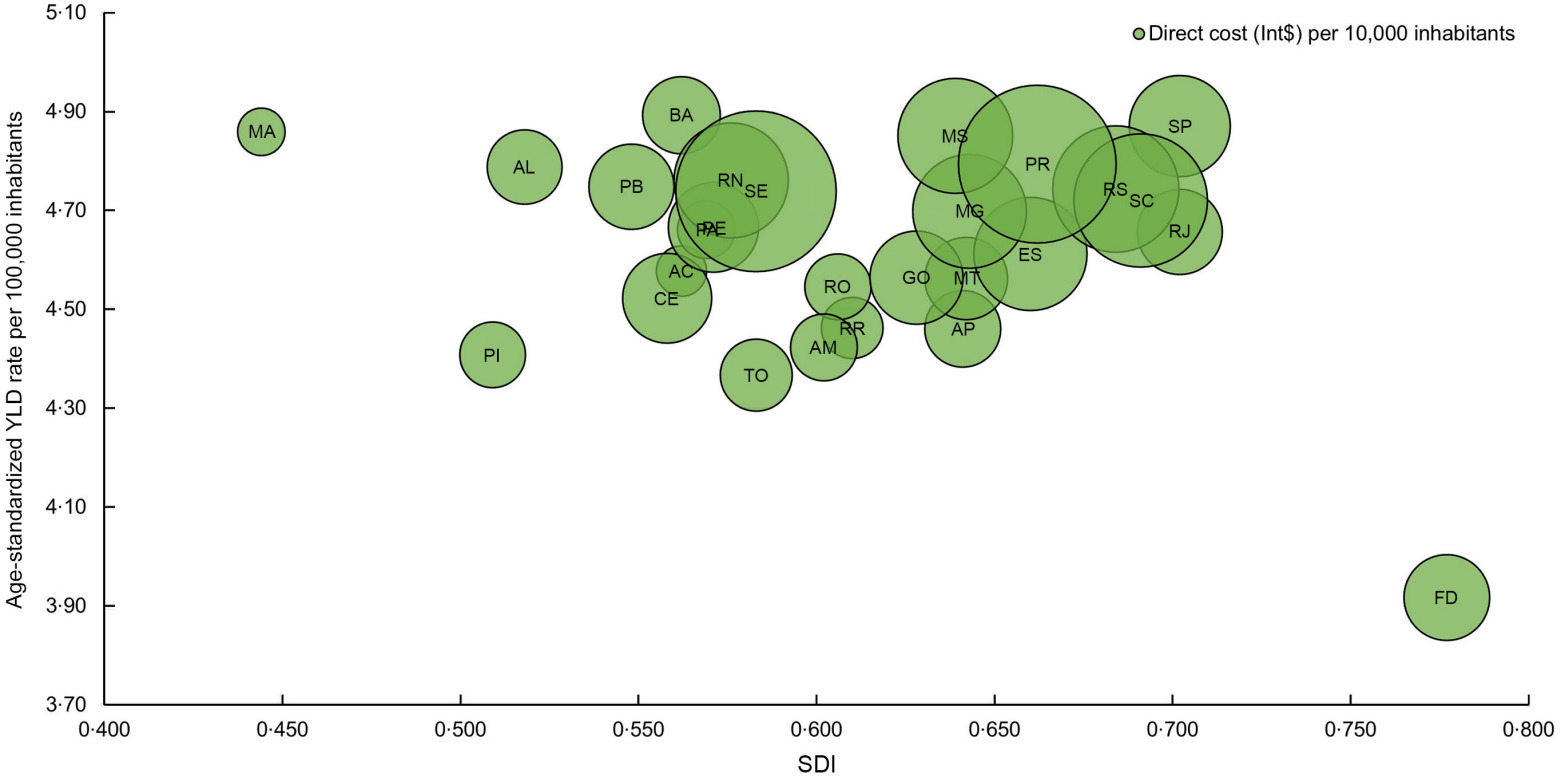
Relationship between the disease burden and economic costs (Int$) to the Unified Health System in Brazil of IHD attributable to trans-fatty acid consumption and the SDI by state, 2019. AC: Acre; AP: Amapá; AM: Amazonas; PA: Pará; RO: Rondônia; RR: Roraima; TO: Tocantins; AL: Alagoas; BA: Bahia; CE: Ceará; MA: Maranhão; PB: Paraíba; PE: Pernambuco; PI: Piauí; RN: Rio Grande do Norte; SE: Sergipe; FD: Federal District; GO: Goiás; MT: Mato Grosso; MS: Mato Grosso do Sul; ES: Espírito Santo; MG: Minas Gerais; RJ: Rio de Janeiro; SP: São Paulo; PR: Paraná; RS: Rio Grande do Sul; SC: Santa Catarina; SDI: socio-demographic index; Int$: international dollar; YLD, years lived with disability

Figure 4 (see online supplementary material, Supplemental Tables S4, S7 and S8) shows the relationship between disease burden and economic costs to the SUS of IHD attributable to TFA consumption and the SDI by state. First, it is noteworthy to highlight that the states in the North and Northeast have the lowest SDI compared to their counterparts in the South, Southeast and Central-West. Overall, populations from the Northeast and North regions, with the lowest SDI, had fewer YLD and spent less, except for Sergipe and Rio Grande do Norte, which presented higher expenditures. In contrast, Maranhão had low expenditure and high YLD. Of note is the trend of increasing costs as the SDI increases, as shown in the states from the South, Southeast and Central-West regions, except for the Federal District.

The results should be interpreted with attention because the 95 % UI range incorporates the uncertainty of the parameters. The broad UI do not allow the identification of differences between the states.

## Discussion

This study revealed that IHD attributable to TFA consumption in Brazil contributed to many years of healthy life loss and represented an enormous direct cost to the country’s public health system. Adhering to the lack of TFA intake (as recommended by GBD) would have avoided approximately 11 166 YLD and saved Int$ 54·5 million (R$ 124·4 million) for the country in 2019. The highest costs were for hospitalisations, males and the population aged ≥ 50 years. Our results also provide insights into these burdens across the country, highlighting the highest crude YLD rate in states from the South and Southeast and a similar pattern in age-standardised YLD rates over the country with some heterogeneity regarding the costs with states from the Northeast and South counting with the bulk of these costs. However, it seems that as the SDI increases, expenditures increase.

The disease burden of IHD attributable to TFA is high in the Brazilian population. Of the total YLD due to IHD in Brazil, the consumption of TFA contributes significantly to this burden (7·6 %, the PAF for both sexes, all ages, in 2019)^(17)^. Deaths and disability-adjusted life years attributable to TFA decreased in the country between 1990 and 2019, but diets high in TFA rose some positions in the ranking in this period^(5)^. In addition, age-standardised YLD rates of CVD attributable to dietary risks have increased over the last 30 years^(3)^. These estimates, in part, reflect improvements in CVD diagnosis and control^(21)^. They can also be explained by the higher TFA intake in Brazil, 1·1 %^(22)^ and 1·4 %^(23)^ of the total daily energy intake in years before 2019, not complying with the WHO recommendations of a maximum of 1 % total daily energy intake^(9)^.

More recent research shows a consumption of TFA between 0·70 % and 0·75 % of the total daily energy for the Brazilian population, with stabilisation of values between 2008 and 2009, and 2017 and 2018^(24,25)^. Despite this, a scenario considering the elimination of PHO in Brazil, the largest source of TFA in the diet, would contribute to three to five times fewer deaths and costs because of premature deaths compared with limiting the TFA content in foods^(13)^. These estimates and our results align with the current policies regarding TFA in Brazil (following an international agenda), which aimed for a 2 % reduction in TFA from total fats by 2021 with complete elimination starting in January 2023^(11)^. Besides that, it is worth mentioning that Brazil has been implementing policies to reduce TFA intake over time. In 2003, the requirement for trans-fat disclosure on nutritional labels was established as mandatory, succeeded by the zero TFA claims^(26)^. Subsequently, in 2008, the national food industries voluntarily committed to the Ministry of Health’s action plan for reducing iTFA through the Declaration of Rio de Janeiro^(27)^.

The disability caused by CVD contributes to a substantial financial cost to the Brazilian health system^(28)^. However, the impact of dietary risk factors such as sodium^(29)^, sugar-sweetened beverages^(30)^ and processed meat^(31)^ has recently been highlighted in Brazil. To the best of our knowledge, our study provides the first cost analysis of the disease attributable to TFA in SUS. These estimates regarding diets high in TFA are insightful for policymakers when considering the potential of health policies and the savings in the direct costs for the health system, as already shown for Australia^(32)^, or even when considering the savings associated with the years of productivity lost in Brazil (US$ 166·7 million)^(13)^.

Although our study included TFA from all sources (ruminant and industrial) and it would be difficult to exclude total TFA from the diet, it is known that food and diet contain more iTFA^(8,33)^. While beef, lamb and dairy products comprise 2–9 % of the total fatty acids such as TFA, PHO comprises up to 60 % TFA. The reduction in iTFA could have a substantial impact on decreasing the health burden in Brazil^(13)^.

The cost analysis by sex and age revealed a higher burden of IHD attributable to TFA in men and individuals aged ≥ 50 years. Higher exposure to risk factors, such as an unhealthy diet, and reduced concern towards disease prevention and use of services for the detection and control may promote higher disability in male individuals^(5,21,25)^. Additionally, the fact that may explain the economic burden of IHD related to TFA consumption in the adult and elderly populations is the suboptimal diet during childhood and adolescence^(25)^, which may suggest that the promotion of a healthy diet should begin in the early stages of life. Besides the lifestyle changes brought on by urbanisation and globalisation, which affect diet and physical activity patterns, the rapid population growth due to the increase in life expectancy can help explain the significant impact of health problems at the oldest ages^(34)^. Thus, although NCD may appear early in life, they progress commonly slowly, starting at younger ages but manifesting during adulthood, having a cumulative effect with advancing age^(35)^. Then, even though TFA consumption may decrease with age^(25)^, the effects of TFA consumption may accumulate over a lifetime, and the burden of IHD disease increases with age. Furthermore, we hypothesise that although ultra-processed foods are sources of iTFA, some products, such as soft drinks, certain types of bread and cookies, may contain little or no TFA. Additionally, we propose that other factors, such as age itself, may have more impact on the disease burden among older people than iTFA (and consequently, ultra-processed foods). Perhaps these factors could explain why the burden of IHD does not follow the intake of ultra-processed foods with increasing age in Brazil.

Another important finding of our study is the higher costs for hospitalisations compared to outpatient care. Hospitalisations due to CVD resulted in the highest expenditure related to hospital admissions in Brazil^(28)^. These results suggest that disease prevention contributes to the reduction of more complex and specialised treatments. However, new efforts are still needed to improve CVD prevention and control in the country with the aim of reducing risk factors^(2,3)^.

In terms of differences in YLD by state, this may reflect the complex relationship among diet, socio-demographic characteristics, interventions and health services targeting CVD, which reflects regional inequalities and inequities in health. Moreover, this study advances the current understanding of the distribution of the direct costs of TFA on IHD, providing information stratified by states. Evaluating the absolute number, we found that states from the South and Southeast regions, with the highest SDI, presented the highest costs, mainly São Paulo, possibly because of their large population size and ageing in these states^(36)^. Therefore, the increasing absolute number of incidents and prevalent cases of IHD means that national health systems need to address more IHD-related procedures to detect and control diseases as the trend continues^(3)^. The rising costs can result in substantial increases in the costs of public healthcare services that could be avoided by food and regulatory policies focused on interventions to reduce TFA consumption and then reduce IHD, save related expenditures and promote the population’s well-being.

After population size was considered in our analysis, the states with the highest direct costs to the SUS per 10 000 inhabitants were from the Northeast and South. Notably, these findings need to be better understood, but states with the lowest and highest SDI are experiencing increased costs of IHD attributable to TFA. Nonetheless, it seems that there is a general pattern of increasing expenditures as the SDI increases. The states with the highest SDI also presented high YLD rates attributable to TFA. Populations with better socioeconomic conditions (such as those from the South and Southeast in Brazil) consume more fat and ultra-processed foods^(23,25,37)^ and have a broader network of secondary and tertiary care services^(38)^ (accounted for in the study and which have a high cost). Therefore, if the population accesses these services more, they can contribute to an increase in expenditure. In contrast, although the Brazilian population in states with low SDI has been increasing ultra-processed food consumption over time^(37)^, studies have already shown individuals in low SDI regions in Brazil and worldwide presenting pronounced poor eating habits and fewer investments in medical care^(38–40)^. In addition, the Brazilian states with the lowest private health insurance coverage have the lowest cost, further revealing the inequalities in the country^(41)^.

Reflecting this hypothesis regarding the regional inequalities and the public financing of hospitalisations related to CVD in Brazil, a study showed that in terms of per capita for people aged ≥ 40 years, the expenditures were considerably lower in states from the North (US$ 6·07) and Northeast (US$ 10·28) than in the southern states (US$ 20·32), even with CVD mortality varying little across the regions^(34)^. Additional potential explanations for the observed lower costs in the North and Northeast states could be attributed to their younger populations^(36)^ and lower YLD. Another possible explanation is the high coverage of primary healthcare in these regions, mainly in the Northeast, perhaps contributing to more access to preventive care instead of curative care^(42,43)^.

The Federal District, with a high SDI and small disease and economic burden, seems closer to a more economically developed state with controlled outcome and risk factor actions, different from Maranhão, with a high disease burden but low expenditures to control them. Regarding social conditions, our results align with the nationwide findings in 2019, wherein the Federal District exhibited more favourable social indicators, while Maranhão demonstrated less favourable ones^(44)^. In Brazil, many NCD have social gradients towards the most socially vulnerable populations^(21)^. Despite the poorest population experiencing worse health outcomes and having more difficult access, especially concerning secondary and tertiary care, progress made by the SUS over the past 30 years has led to improved outcomes and reduced health inequities^(45)^.

The SUS is the Brazilian public health system responsible for providing free access to healthcare at all levels for the entire Brazilian population^(14)^. In 2019, approximately 76 % of the population exclusively depended on the SUS to access medical services^(46)^. The SUS plays a crucial role as the primary healthcare provider for the poorest population and those with limited access to private health insurance^(45)^. Understanding the economic burden allows for prioritising policies, interventions and allocation of health resources according to the budgetary constraints of this system^(2)^. Therefore, in addition to highlighting the disease burden on the population and the potential cost savings for the SUS if iTFA consumption is zero, our findings also contribute to optimising healthcare investments and providing guidance on resource allocation at the subnational level. Moreover, it should be mentioned that considering the health promotion model, the best quality service would prevent illness by promoting access to a healthy diet, a more cost-effective action^(47)^. Notably, our results enable better management of healthcare and related costs across the country according to the TFA-attributable disease burden and socio-demographic conditions but, most importantly, can contribute to the decisions of public policies to target iTFA to reduce disease and economic burden more effectively.

This study had some limitations which are worth noting. Although secondary data sources are essential for public health, the disease may be misclassified. In addition, disease classification and data depend on access to the diagnosis and awareness of the correct completion of information systems used for epidemiological surveillance. For example, subnational data can have different characteristics. Also, data related to states can result in imprecise estimates because most epidemiological data sources, such as surveys, are disaggregated to regions and not to states. In GBD, the impact of dietary risk factors on disease outcomes primarily stems from meta-analyses of prospective observational studies. While adjustments for confounding variables such as age, sex, smoking and physical activity have been made in many cases, residual confounding remains a potential concern^(15)^. GBD uses many strategies to improve and compare data; however, biases are inevitable because many databases are used^(15)^.

Another limitation is that the most recent Brazilian consumption surveys^(25)^ were not considered in GBD 2019. On the contrary, the data were obtained from a global market information database on sales. For example, this could weaken our conclusions on differences according to age since IHD increases with age, whereas ultra-processed food consumption (the dietary sources of iTFA) decreases with age in Brazil^(25)^. However, as mentioned before, ultra-processed consumption has cumulative effects on NCD; some ultra-processed foods consumed by this population may have little or no iTFA or may even have a lesser impact on the disease burden during ageing. Although our study included TFA from all sources (natural and industrial), which would make it difficult to exclude total TFA from the diet, it is known that food and diet contain more iTFA^(8,33)^.

Regarding the costs of IHD attributable to TFA consumption, it is worth mentioning that our findings can be underestimated because we only considered public healthcare expenditure and direct costs. The Brazilian publicly available data on private health do not include individualised data and ICD codes*—*essential in our study to determine the TFA-attributable burden. Moreover, we do not include the costs of primary healthcare and other direct costs, such as medications to use at home provided by the SUS, rehabilitation, payment for caregivers and transportation of the patient to the health facility. Finally, using PAF may not represent the picture of disease and economic burden because of limitations, for example, limited or inaccurate data, complexity of calculations and assumption of homogeneity of effects among different populations. These uncertainties can propagate through the PAF calculation, affecting the reliability of the results. However, this is a well-established approach to quantify the contribution of specific risk factors to the disease and economic burden in a population, inform public health priorities and have been used in other similar studies^(30,31,48–50)^.

Our study has several strengths. The originality and strength of this work lie in the disease and economic burden assessment related to the consumption of TFA in Brazil, especially in its twenty-seven states. To the best of our knowledge, this study is the first to provide evidence on disability and direct costs to the Brazilian public health system attributable to TFA at the subnational level. We revealed the potential health benefits to the Brazilian population and cost savings to the SUS if TFA consumption was reduced. Moreover, we further investigated sex, age groups and SDI, wherein this reduction could result in the highest cost savings. As a perspective, we also encourage future analyses incorporating other variables not included in this study, such as differences between the area of residence (urban or rural), access to health services, income, education and race. Another strength is that we considered the risk-outcome pair, relative risks, PAF and uncertainty estimated by GBD, which has a robust, standardised and updated methodology^(1,6)^. Lastly, this study strengthens the awareness of policymakers and other stakeholders towards TFA’s global public health agenda^(9)^ based on evidence found in the country. These estimates help draw attention to the international agenda accepted by Brazil, aiming to reduce and eliminate iTFA globally by 2023^(9,10)^. Therefore, these estimates reinforce that the country should prioritise the elimination of iTFA as already in progress, prioritising the population’s health in the face of long-term policies^(13)^.

### Conclusions

This study showed that IHD attributable to TFA consumption contributed to high disability in the population and costs to the Brazilian health system in 2019. Overall, heterogeneity in the economic burden across Brazilian states is observed, which reveals inequalities regarding disease expenditures over the country; however, there is an indication that as their SDI increases, direct costs also increase. Thus, our findings reinforce that more stringent policies, such as iTFA elimination, as suggested by the international agenda, can contribute to health gains and economic savings, in addition to reducing subnational inequalities by prioritising the allocation of resources and sustainability of the SUS.

## Supporting information

Parajára et al. supplementary material Parajára et al. supplementary material

## Supplementary material

For supplementary material accompanying this paper, visit https://doi.org/10.1017/S1368980024001101.
