# Supplementary material for: Disability and costs of IHD attributable to the consumption of trans-fatty acids in Brazil
Source: Public Health Nutr. 2024 May 10;27(1):e132. doi: 10.1017/S1368980024001101 (PMC11112431; doi:10.1017/S1368980024001101)
Supplement: Parajára et al. supplementary material [file S1368980024001101sup001.pdf]

Disability and costs of ischemic heart disease attributable to the consumption of trans fatty acids in Brazil

Supplementary Material

This document comprises a figure to illustrate the Brazilian states and regions; tables with data used in the Global Burden of Disease (GBD) 2019 Study, coming from the GBD 2019 Risk Factors Collaborators Supplementary Materials; and the values used in the findings of this study, with their respective 95% uncertainty intervals (95% UIs), coming from the Exchange website (GHDx; https://ghdx.healthdata.org/) via GBD Results Tool.

Summary

Table S1 Relative risks by age and sex for ischemic heart disease attributable to diets high in trans fatty acids. 2

Table S2 Sources of the relative risks by age and sex for ischemic heart disease attributable to diets high in trans fatty acids. 3

Figure S1 Description of Brazilian states and regions. 4

Table S3 Population attributable fraction (PAF), in %, for years lived with disability due to ischemic heart disease attributable to a diet high in trans fatty acids in Brazil in 2019 stratified by sex, age, and state. 5

Table S4 Number and rates of years lived with disability, per 100,000, for ischemic heart disease attributable to trans fatty acids consumption in Brazil and states, 2019. 25

Table S5 The direct cost (Int$) to the Unified Health System of mediators between trans fatty acids consumption and ischemic heart disease, LDL-c, and systolic blood pressure in Brazil by type of procedure, 2019. 26

Table S6 The direct cost (Int$) of ischemic heart disease attributable to the trans fatty acids consumption to the Unified Health System in Brazil by states, 2019. 27

Table S7 Population and the direct cost (Int$) per 10,000 inhabitants of ischemic heart disease attributable to the trans fatty acids consumption to the Unified Health System in Brazil by states, 2019. 28

Table S8 Socio-demographic index values from Brazil and its states in 2019. 29

References 30

Table S1 Relative risks by age and sex for ischemic heart disease attributable to diets high in trans fatty acids.

Risk-outcome	Category/ Unit	Morbidity/ Mortality	Sex	Age (years)														
				25-29	30-34	35-39	40-44	45-49	50-54	55-59	60-64	65-69	70-74	75-79	80-84	85-89	90-94	95+
**Diet high in trans fatty acids**																		
Ischemic heart disease	0% Energy/day	Both	Both	1.00 (1.00, 1.00)	1.00 (1.00, 1.00)	1.00 (1.00, 1.00)	1.00 (1.00, 1.00)	1.00 (1.00, 1.00)	1.00 (1.00, 1.00)	1.00 (1.00, 1.00)	1.00 (1.00, 1.00)	1.00 (1.00, 1.00)	1.00 (1.00, 1.00)	1.00 (1.00, 1.00)	1.00 (1.00, 1.00)	1.00 (1.00, 1.00)	1.00 (1.00, 1.00)	1.00 (1.00, 1.00)
Ischemic heart disease	1% Energy/day	Both	Both	1.28 (1.01, 1.38)	1.28 (1.01, 1.38)	1.28 (1.01, 1.38)	1.23 (1.01, 1.38)	1.22 (1.00, 1.29)	1.20 (1.00, 1.27)	1.17 (1.00, 1.23)	1.15 (1.00, 1.20)	1.13 (1.00, 1.17)	1.12 (1.00, 1.16)	1.11 (1.00, 1.14)	1.11 (1.00, 1.14)	1.11 (1.00, 1.14)	1.11 (1.00, 1.14)	1.11 (1.00, 1.14)
Ischemic heart disease	2% Energy/day	Both	Both	1.37 (1.01, 1.54)	1.37 (1.01, 1.54)	1.37 (1.01, 1.54)	1.30 (1.01, 1.43)	1.28 (1.01, 1.41)	1.26 (1.01, 1.37)	1.22 (1.01, 1.31)	1.19 (1.00, 1.27)	1.17 (1.00, 1.24)	1.15 (1.00, 1.21)	1.14 (1.00, 1.20)	1.14 (1.00, 1.20)	1.14 (1.00, 1.20)	1.14 (1.00, 1.20)	1.14 (1.00, 1.20)
Ischemic heart disease	3% Energy/day	Both	Both	1.38 (1.01, 1.54)	1.38 (1.01, 1.54)	1.38 (1.01, 1.54)	1.30 (1.01, 1.43)	1.29 (1.01, 1.37)	1.26 (1.01, 1.41)	1.22 (1.01, 1.31)	1.19 (1.00, 1.27)	1.17 (1.00, 1.24)	1.15 (1.00, 1.21)	1.14 (1.00, 1.20)	1.14 (1.00, 1.20)	1.14 (1.00, 1.20)	1.14 (1.00, 1.20)	1.14 (1.00, 1.20)
Ischemic heart disease	4% Energy/day	Both	Both	1.38 (1.01, 1.54)	1.38 (1.01, 1.54)	1.38 (1.01, 1.54)	1.30 (1.01, 1.43)	1.29 (1.01, 1.37)	1.26 (1.01, 1.41)	1.22 (1.01, 1.31)	1.19 (1.00, 1.27)	1.17 (1.00, 1.24)	1.15 (1.00, 1.21)	1.14 (1.00, 1.20)	1.14 (1.00, 1.20)	1.14 (1.00, 1.20)	1.14 (1.00, 1.20)	1.14 (1.00, 1.20)

Source: Supplement to GBD 2019 Risk Factors Collaborators, 2019.

Table S2 Sources of the relative risks by age and sex for ischemic heart disease attributable to diets high in trans fatty acids.

Data Type	Title	Citation
Scientific literature	Intake of fatty acids and risk of coronary heart disease in a cohort of Finnish men. The Alpha-Tocopherol, Beta-Carotene Cancer Prevention Study	Pietinen P, Ascherio A, Korhonen P, Hartman AM, Willett WC, Albanes D, Virtamo J. Intake of fatty acids and risk of coronary heart disease in a cohort of Finnish men. The Alpha-Tocopherol, Beta-Carotene Cancer Prevention Study. Am J Epidemiol. 1997; 145(10): 876–87.
Scientific literature	Dietary fat intake and risk of coronary heart disease in women: 20 years of follow-up of the nurses' health study	Oh K, Hu FB, Manson JE, Stampfer MJ, Willett WC. Dietary fat intake and risk of coronary heart disease in women: 20 years of follow-up of the nurses' health study. Am J Epidemiol. 2005; 161(7): 672–9.
Scientific literature	Association between trans fatty acid intake and 10-year risk of coronary heart disease in the Zutphen Elderly Study: a prospective population-based study	Oomen CM, Ocké MC, Feskens EJ, van Erp-Baart MA, Kok FJ, Kromhout D. Association between trans fatty acid intake and 10-year risk of coronary heart disease in the Zutphen Elderly Study: a prospective population-based study. Lancet. 2001; 357(9258): 746–51.
Scientific literature	Dietary fat and risk of coronary heart disease in men: cohort follow up study in the United States	Ascherio A, Rimm EB, Giovannucci EL, Spiegelman D, Stampfer M, Willett WC. Dietary fat and risk of coronary heart disease in men: cohort follow up study in the United States. BMJ. 1996; 313(7049): 84–90.
Scientific literature	Intake of trans fatty acids and risk of coronary heart disease among women	Willett WC, Stampfer MJ, Manson JE, Colditz GA, Speizer FE, Rosner BA, Sampson LA, Hennekens CH. Intake of trans fatty acids and risk of coronary heart disease among women. Lancet. 1993; 341(8845): 581-5.
Scientific literature	Dietary fat intake and risk of coronary heart disease: the Strong Heart Study	Xu J, Eilat-Adar S, Loria C, Goldbourt U, Howard BV, Fabsitz RR, Zephier EM, Mattil C, Lee ET. Dietary fat intake and risk of coronary heart disease: the Strong Heart Study. Am J Clin Nutr. 2006; 84(4): 894-902.
Scientific literature	Intake of ruminant trans fatty acids and risk of coronary heart disease	Jakobsen MU, Overvad K, Dyerberg J, Heitmann BL. Intake of ruminant trans fatty acids and risk of coronary heart disease. Int J Epidemiol. 2008; 37(1): 173-82.
Scientific literature	A prospective study of intake of trans-fatty acids from ruminant fat, partially hydrogenated vegetable oils, and marine oils and mortality from CVD	Laake I, Pedersen JI, Selmer R, Kirkhus B, Lindman AS, Tverdal A, Veierød MB. A prospective study of intake of trans-fatty acids from ruminant fat, partially hydrogenated vegetable oils, and marine oils and mortality from CVD. Br J Nutr. 2012; 108(4): 743-54.
Scientific literature	Dietary fatty acids and risk of coronary heart disease in men: the Kuopio Ischemic Heart Disease Risk Factor Study	Virtanen JK, Mursu J, Tuomainen TP, Voutilainen S. Dietary fatty acids and risk of coronary heart disease in men: the Kuopio Ischemic Heart Disease Risk Factor Study. Arterioscler Thromb Vasc Biol. 2014; 34(12): 2679-87.
Scientific literature	Low-fat dietary pattern and risk of cardiovascular disease: the Women's Health Initiative Randomized Controlled Dietary Modification Trial	Howard BV, Van Horn L, Hsia J, Manson JE, Stefanick ML, Wassertheil-Smoller S, Kuller LH, LaCroix AZ, Langer RD, Lasser NL, Lewis CE, Limacher MC, Margolis KL, Mysiw WJ, Ockene JK, Parker LM, Perri MG, Phillips L, Prentice RL, Robbins J, Rossouw JE, Sarto GE, Schatz IJ, Snetselaar LG, Stevens VJ, Tinker LF, Trevisan M, Vitolins MZ, Anderson GL, Assaf AR, Bassford T, Beresford SA, Black HR, Brunner RL, Brzyski RG, Caan B, Chlebowski RT, Gass M, Granek I, Greenland P, Hays J, Heber D, Heiss G, Hendrix SL, Hubbell FA, Johnson KC, Kotchen JM. Low-fat dietary pattern and risk of cardiovascular disease: the Women's Health Initiative Randomized Controlled Dietary Modification Trial. JAMA. 2006; 295(6): 655-66.

Source: Global Burden of Disease Collaborative Network, 2020.

Figure S1 Description of Brazilian states and regions.

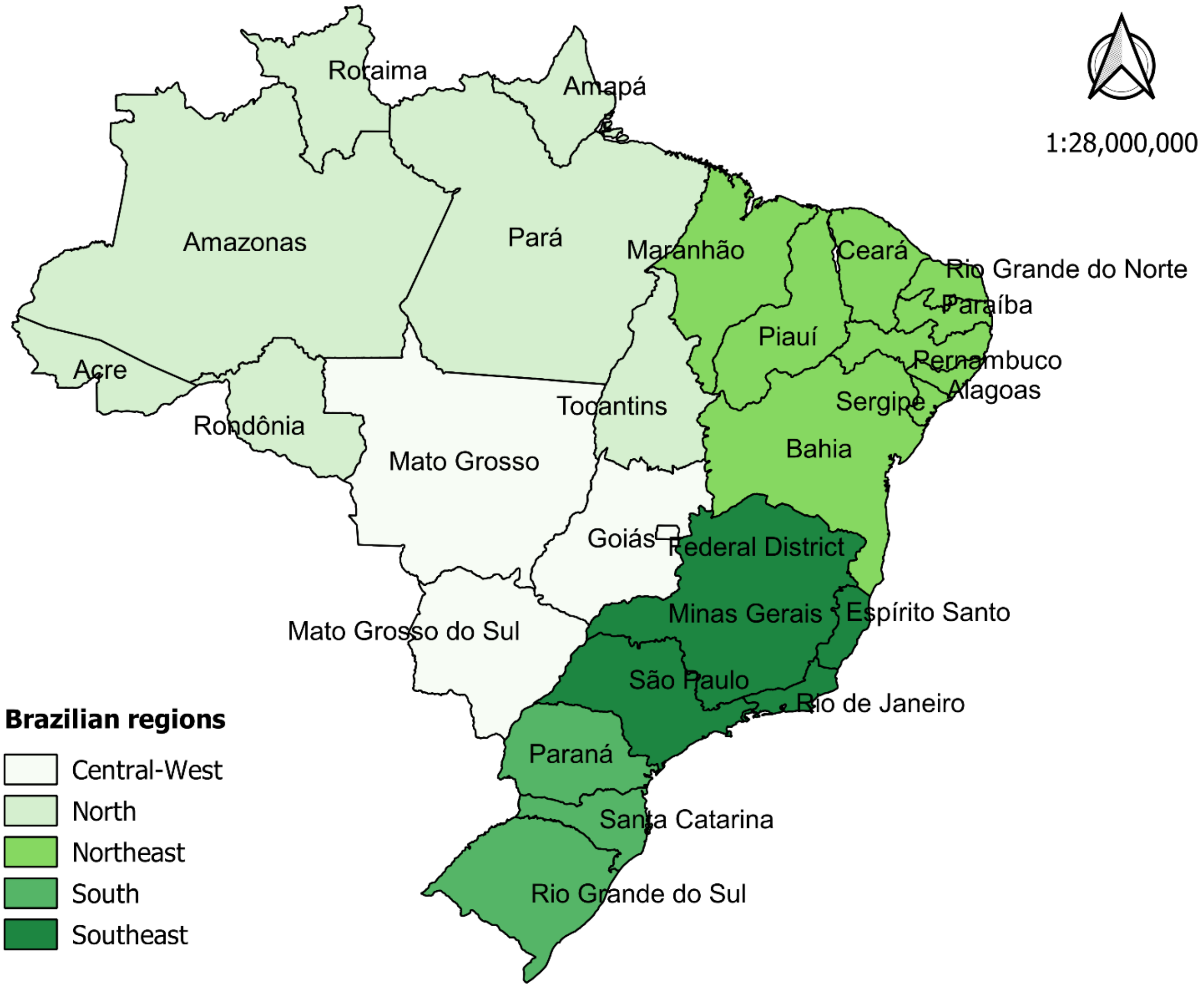

Source: Elaborated by the authors.

Table S3 Population attributable fraction (PAF), in %, for years lived with disability due to ischemic heart disease attributable to a diet high in trans fatty acids in Brazil in 2019 stratified by sex, age, and state.

Location (state)	Sex	Age	Value (%)	Lower value (%)	Upper value (%)
Acre	Female	25 to 29	12.703279	0.8823757	19.911528
Acre	Female	30 to 34	12.701975	1.0276515	19.826644
Acre	Female	35 to 39	12.819989	1.061423	20.116618
Acre	Female	40 to 44	10.836655	0.7673544	17.265141
Acre	Female	45 to 49	10.594918	0.8929875	16.933833
Acre	Female	50 to 54	9.840121	0.8524806	15.18795
Acre	Female	55 to 59	8.6727083	0.7500942	13.797859
Acre	Female	60 to 64	7.8029752	0.6588368	12.163568
Acre	Female	65 to 69	7.3067453	0.6120971	11.600307
Acre	Female	70 to 74	6.7703497	0.6424641	10.70735
Acre	Female	75 to 79	6.3918367	0.5951335	10.098824
Acre	Female	80 to 84	6.4923605	0.6293616	10.181902
Acre	Female	85 to 89	6.4934737	0.6176809	10.232859
Acre	Female	90 to 94	6.5470458	0.6092084	10.266571
Acre	Female	95 plus	6.4968921	0.6113727	10.13054
Acre	Male	25 to 29	12.389287	0.7614191	19.614685
Acre	Male	30 to 34	12.440055	0.8859687	20.098409
Acre	Male	35 to 39	12.631519	0.8362683	19.670441
Acre	Male	40 to 44	10.757436	0.6941409	17.210321
Acre	Male	45 to 49	10.381172	0.7209401	16.511095
Acre	Male	50 to 54	9.8259875	0.657985	15.855681
Acre	Male	55 to 59	8.64102	0.6259585	13.908195
Acre	Male	60 to 64	7.698476	0.5637391	12.08061
Acre	Male	65 to 69	7.2385131	0.5984635	11.531329
Acre	Male	70 to 74	6.7466068	0.5498317	10.62459
Acre	Male	75 to 79	6.2848597	0.5068833	9.8123241
Acre	Male	80 to 84	6.4691048	0.5752292	10.467014
Acre	Male	85 to 89	6.4519814	0.5553795	10.254434
Acre	Male	90 to 94	6.4341396	0.5536779	10.027674
Acre	Male	95 plus	6.4492191	0.5110914	10.475585
Alagoas	Female	25 to 29	12.738801	0.9202911	19.483742
Alagoas	Female	30 to 34	12.717413	0.8963478	20.583945
Alagoas	Female	35 to 39	12.818413	1.0099396	19.888117
Alagoas	Female	40 to 44	10.804616	0.8133035	16.910464
Alagoas	Female	45 to 49	10.504278	0.9250377	16.320281
Alagoas	Female	50 to 54	9.8544121	0.8678967	15.186156
Alagoas	Female	55 to 59	8.7546662	0.799136	14.00047
Alagoas	Female	60 to 64	7.8264769	0.7093762	12.401569

Alagoas	Female	65 to 69	7.21111	0.6868638	11.299774
Alagoas	Female	70 to 74	6.7564594	0.6339459	10.482918
Alagoas	Female	75 to 79	6.3713142	0.6085525	10.203708
Alagoas	Female	80 to 84	6.4348694	0.6695495	10.224354
Alagoas	Female	85 to 89	6.5102467	0.6141417	10.275228
Alagoas	Female	90 to 94	6.4764826	0.6227479	9.9775255
Alagoas	Female	95 plus	6.4844988	0.619292	10.13988
Alagoas	Male	25 to 29	12.496543	0.805843	20.01545
Alagoas	Male	30 to 34	12.382761	0.8386005	19.565573
Alagoas	Male	35 to 39	12.636754	0.8243969	19.911569
Alagoas	Male	40 to 44	10.671143	0.7375182	16.951846
Alagoas	Male	45 to 49	10.391436	0.6944729	16.525164
Alagoas	Male	50 to 54	9.7655717	0.6740929	15.684306
Alagoas	Male	55 to 59	8.6384623	0.6225753	14.041794
Alagoas	Male	60 to 64	7.776259	0.6580551	12.251282
Alagoas	Male	65 to 69	7.2006977	0.5857865	11.459341
Alagoas	Male	70 to 74	6.7407339	0.5452483	10.627067
Alagoas	Male	75 to 79	6.2770892	0.551431	9.9600262
Alagoas	Male	80 to 84	6.4398844	0.5721588	10.132132
Alagoas	Male	85 to 89	6.4290908	0.5871587	10.147519
Alagoas	Male	90 to 94	6.4011541	0.5376177	9.9758766
Alagoas	Male	95 plus	6.4103288	0.5272778	10.507625
Amapá	Female	25 to 29	12.698076	0.8790347	19.828492
Amapá	Female	30 to 34	12.660252	0.9894442	19.619144
Amapá	Female	35 to 39	12.886141	0.9711724	20.319281
Amapá	Female	40 to 44	10.760811	0.9340308	16.99542
Amapá	Female	45 to 49	10.598596	0.9299066	16.348367
Amapá	Female	50 to 54	9.8330871	0.8410432	15.496651
Amapá	Female	55 to 59	8.7327405	0.6894661	13.9855
Amapá	Female	60 to 64	7.8486328	0.7162064	12.479123
Amapá	Female	65 to 69	7.3258057	0.6553054	11.606624
Amapá	Female	70 to 74	6.7223326	0.5877609	10.566949
Amapá	Female	75 to 79	6.4166165	0.651068	10.072424
Amapá	Female	80 to 84	6.4321368	0.6643333	9.9189453
Amapá	Female	85 to 89	6.5143628	0.6285332	10.327269
Amapá	Female	90 to 94	6.5190822	0.5793982	10.256781
Amapá	Female	95 plus	6.4441545	0.6005626	9.9807652
Amapá	Male	25 to 29	12.469286	0.7571761	19.652615
Amapá	Male	30 to 34	12.514116	0.7839352	20.662636
Amapá	Male	35 to 39	12.600302	0.7556849	19.927011
Amapá	Male	40 to 44	10.685332	0.7148108	17.167544
Amapá	Male	45 to 49	10.376567	0.7331341	16.775124
Amapá	Male	50 to 54	9.8615141	0.7079222	16.022383

Amapá	Male	55 to 59	8.6026261	0.5898035	13.625596
Amapá	Male	60 to 64	7.8013494	0.5812314	12.431241
Amapá	Male	65 to 69	7.2375422	0.6073667	11.788653
Amapá	Male	70 to 74	6.7200842	0.5431875	10.723284
Amapá	Male	75 to 79	6.2604343	0.5695744	9.6416472
Amapá	Male	80 to 84	6.4754639	0.5274185	10.315564
Amapá	Male	85 to 89	6.4216845	0.5887392	10.220675
Amapá	Male	90 to 94	6.3722237	0.587823	9.833161
Amapá	Male	95 plus	6.3933598	0.5401719	10.438134
Amazonas	Female	25 to 29	12.633357	0.8866612	19.73298
Amazonas	Female	30 to 34	12.660225	0.979937	19.87447
Amazonas	Female	35 to 39	12.871459	1.0518909	19.963816
Amazonas	Female	40 to 44	10.864718	0.7989151	17.377149
Amazonas	Female	45 to 49	10.556535	0.88062	16.468645
Amazonas	Female	50 to 54	9.7571585	0.7667506	14.979074
Amazonas	Female	55 to 59	8.6748364	0.8235069	13.632485
Amazonas	Female	60 to 64	7.8223909	0.7413032	12.35129
Amazonas	Female	65 to 69	7.2989627	0.7038054	11.522491
Amazonas	Female	70 to 74	6.726345	0.642495	10.496969
Amazonas	Female	75 to 79	6.4362035	0.6519926	9.9493503
Amazonas	Female	80 to 84	6.4410769	0.6274959	10.15442
Amazonas	Female	85 to 89	6.4845442	0.6107241	10.051163
Amazonas	Female	90 to 94	6.5273631	0.6298924	10.314947
Amazonas	Female	95 plus	6.5026718	0.6278543	10.123923
Amazonas	Male	25 to 29	12.550479	0.7540356	19.564117
Amazonas	Male	30 to 34	12.537901	0.7600023	20.227446
Amazonas	Male	35 to 39	12.602955	0.7576137	20.418574
Amazonas	Male	40 to 44	10.761615	0.7853699	17.300197
Amazonas	Male	45 to 49	10.454052	0.6559185	16.551262
Amazonas	Male	50 to 54	9.8090444	0.6568507	15.289758
Amazonas	Male	55 to 59	8.6432194	0.6211252	14.254891
Amazonas	Male	60 to 64	7.797989	0.5805676	12.359604
Amazonas	Male	65 to 69	7.1978567	0.6431222	11.39304
Amazonas	Male	70 to 74	6.7140414	0.5582737	10.63406
Amazonas	Male	75 to 79	6.262552	0.5575037	10.121007
Amazonas	Male	80 to 84	6.4586228	0.603082	10.452327
Amazonas	Male	85 to 89	6.4047151	0.5367926	9.9047498
Amazonas	Male	90 to 94	6.413394	0.5937817	10.153304
Amazonas	Male	95 plus	6.4674356	0.5861233	10.392039
Bahia	Female	25 to 29	12.662341	0.902756	19.219111
Bahia	Female	30 to 34	12.67572	0.9197523	19.654547
Bahia	Female	35 to 39	12.678498	0.925256	19.600179
Bahia	Female	40 to 44	10.831109	0.8219311	16.716792

Bahia	Female	45 to 49	10.546035	0.878314	15.918009
Bahia	Female	50 to 54	9.7584619	0.8515155	15.419502
Bahia	Female	55 to 59	8.6710863	0.7130789	13.539921
Bahia	Female	60 to 64	7.816283	0.7072278	12.012978
Bahia	Female	65 to 69	7.2976546	0.7333939	11.739815
Bahia	Female	70 to 74	6.7190694	0.6710393	10.389537
Bahia	Female	75 to 79	6.4047057	0.6461523	10.013195
Bahia	Female	80 to 84	6.4561179	0.6487255	10.054431
Bahia	Female	85 to 89	6.4669878	0.5699403	9.9219818
Bahia	Female	90 to 94	6.5044186	0.592462	10.102974
Bahia	Female	95 plus	6.4576739	0.5945088	10.018912
Bahia	Male	25 to 29	12.444464	0.7937915	19.056136
Bahia	Male	30 to 34	12.502522	0.7546653	19.900204
Bahia	Male	35 to 39	12.64562	0.7867947	19.927843
Bahia	Male	40 to 44	10.797112	0.7615884	17.441554
Bahia	Male	45 to 49	10.361023	0.7092646	16.683392
Bahia	Male	50 to 54	9.760699	0.7032272	15.446014
Bahia	Male	55 to 59	8.6357075	0.6242358	13.962579
Bahia	Male	60 to 64	7.6839444	0.5803572	11.955745
Bahia	Male	65 to 69	7.2706319	0.6009429	11.633168
Bahia	Male	70 to 74	6.7426195	0.5484454	10.52561
Bahia	Male	75 to 79	6.2502495	0.5069965	9.8169461
Bahia	Male	80 to 84	6.4365224	0.5839244	10.088979
Bahia	Male	85 to 89	6.4645277	0.6046274	10.295876
Bahia	Male	90 to 94	6.4499024	0.5466856	10.128078
Bahia	Male	95 plus	6.4079423	0.5405116	10.051332
Ceará	Female	25 to 29	12.768821	0.8630531	20.405911
Ceará	Female	30 to 34	12.7436	0.8339053	19.641412
Ceará	Female	35 to 39	12.781762	1.0558935	20.202497
Ceará	Female	40 to 44	10.798457	0.8194814	16.886286
Ceará	Female	45 to 49	10.545943	0.8879392	16.228808
Ceará	Female	50 to 54	9.8341397	0.8094008	15.526637
Ceará	Female	55 to 59	8.6755655	0.7503462	13.888349
Ceará	Female	60 to 64	7.843417	0.7044956	12.284912
Ceará	Female	65 to 69	7.2450215	0.6665821	11.372509
Ceará	Female	70 to 74	6.7227222	0.7226976	10.403853
Ceará	Female	75 to 79	6.4246506	0.758476	9.8995559
Ceará	Female	80 to 84	6.4101254	0.5821999	9.7800553
Ceará	Female	85 to 89	6.4855854	0.6429283	10.324592
Ceará	Female	90 to 94	6.498453	0.6248037	10.029145
Ceará	Female	95 plus	6.4572302	0.5989004	9.8624858
Ceará	Male	25 to 29	12.455224	0.7419299	19.491545
Ceará	Male	30 to 34	12.559311	0.8755471	19.945294

Ceará	Male	35 to 39	12.645554	0.8851646	19.65755
Ceará	Male	40 to 44	10.760578	0.7637112	17.313789
Ceará	Male	45 to 49	10.414472	0.7113437	16.537857
Ceará	Male	50 to 54	9.8381657	0.6246148	15.682299
Ceará	Male	55 to 59	8.5923029	0.6329289	13.669968
Ceará	Male	60 to 64	7.7040972	0.6403588	12.140206
Ceará	Male	65 to 69	7.2076099	0.5948277	11.367742
Ceará	Male	70 to 74	6.6881123	0.5735092	10.541964
Ceará	Male	75 to 79	6.2228874	0.5221819	9.8540754
Ceará	Male	80 to 84	6.4511048	0.5333794	10.338797
Ceará	Male	85 to 89	6.4226313	0.5549705	10.1901
Ceará	Male	90 to 94	6.4141421	0.5680379	10.255774
Ceará	Male	95 plus	6.3907674	0.4785346	10.196992
Distrito Federal	Female	25 to 29	12.735566	0.9747424	20.266124
Distrito Federal	Female	30 to 34	12.654798	0.9942549	19.981843
Distrito Federal	Female	35 to 39	12.767298	0.9462905	19.688023
Distrito Federal	Female	40 to 44	10.849735	0.7998915	17.165009
Distrito Federal	Female	45 to 49	10.54507	0.9283817	16.379764
Distrito Federal	Female	50 to 54	9.870101	0.7401426	15.361337
Distrito Federal	Female	55 to 59	8.7003816	0.7823781	13.53336
Distrito Federal	Female	60 to 64	7.8821436	0.7075076	12.334972
Distrito Federal	Female	65 to 69	7.2949117	0.6455215	11.529443
Distrito Federal	Female	70 to 74	6.7542871	0.6423167	10.572908
Distrito Federal	Female	75 to 79	6.4196111	0.6148033	10.074407
Distrito Federal	Female	80 to 84	6.4697458	0.6728064	10.195799
Distrito Federal	Female	85 to 89	6.5257469	0.5938049	10.342485
Distrito Federal	Female	90 to 94	6.48844	0.6335292	10.315363
Distrito Federal	Female	95 plus	6.4696672	0.5700442	10.113409
Distrito Federal	Male	25 to 29	12.495993	0.8017252	19.530949
Distrito Federal	Male	30 to 34	12.498129	0.8210063	19.625387
Distrito Federal	Male	35 to 39	12.757502	0.7962548	20.141754
Distrito Federal	Male	40 to 44	10.762771	0.7265075	16.960673
Distrito Federal	Male	45 to 49	10.407436	0.7135852	16.811479
Distrito Federal	Male	50 to 54	9.7961182	0.6792193	15.774597
Distrito Federal	Male	55 to 59	8.7190625	0.6000113	14.221383
Distrito Federal	Male	60 to 64	7.7448101	0.6019828	12.285579
Distrito Federal	Male	65 to 69	7.2121222	0.5686046	11.483188
Distrito Federal	Male	70 to 74	6.7148235	0.5549606	10.824496
Distrito Federal	Male	75 to 79	6.2963856	0.5543034	9.8447315
Distrito Federal	Male	80 to 84	6.4772	0.5727046	10.3843
Distrito Federal	Male	85 to 89	6.4427114	0.548372	10.584595
Distrito Federal	Male	90 to 94	6.4081871	0.5418083	10.099341
Distrito Federal	Male	95 plus	6.4388086	0.5582976	10.464048

Espírito Santo	Female	25 to 29	12.711039	0.8790806	19.668605
Espírito Santo	Female	30 to 34	12.744404	0.8812083	19.770837
Espírito Santo	Female	35 to 39	12.875006	1.0312702	19.968408
Espírito Santo	Female	40 to 44	10.7508	0.8613701	16.664881
Espírito Santo	Female	45 to 49	10.611663	0.8712554	16.5319
Espírito Santo	Female	50 to 54	9.8070137	0.89268	15.192194
Espírito Santo	Female	55 to 59	8.7183676	0.8915777	13.878637
Espírito Santo	Female	60 to 64	7.7887024	0.7363992	12.350348
Espírito Santo	Female	65 to 69	7.2808401	0.7223401	10.917489
Espírito Santo	Female	70 to 74	6.7779432	0.6789247	10.651991
Espírito Santo	Female	75 to 79	6.4340907	0.6582814	9.9294723
Espírito Santo	Female	80 to 84	6.4475926	0.6604543	10.136518
Espírito Santo	Female	85 to 89	6.4597727	0.6484178	10.08131
Espírito Santo	Female	90 to 94	6.4936267	0.6083264	10.09883
Espírito Santo	Female	95 plus	6.4704003	0.60306	10.266674
Espírito Santo	Male	25 to 29	12.462232	0.8058576	19.414236
Espírito Santo	Male	30 to 34	12.506555	0.8211443	19.89491
Espírito Santo	Male	35 to 39	12.700646	0.830046	20.404532
Espírito Santo	Male	40 to 44	10.702698	0.734375	17.25179
Espírito Santo	Male	45 to 49	10.47805	0.6912204	17.052052
Espírito Santo	Male	50 to 54	9.8278043	0.6465569	15.533035
Espírito Santo	Male	55 to 59	8.6073596	0.6324214	13.656124
Espírito Santo	Male	60 to 64	7.7127584	0.5841005	12.237858
Espírito Santo	Male	65 to 69	7.1812974	0.5560326	11.51647
Espírito Santo	Male	70 to 74	6.7125478	0.5591073	10.671908
Espírito Santo	Male	75 to 79	6.2551116	0.5392536	9.7143116
Espírito Santo	Male	80 to 84	6.4508308	0.5436317	10.116528
Espírito Santo	Male	85 to 89	6.4631364	0.5484957	10.301613
Espírito Santo	Male	90 to 94	6.4474705	0.6038333	10.41455
Espírito Santo	Male	95 plus	6.4452389	0.5691596	10.416685
Goiás	Female	25 to 29	12.64709	0.9640117	19.825245
Goiás	Female	30 to 34	12.675333	0.8548979	19.848924
Goiás	Female	35 to 39	12.730745	1.0286997	19.735786
Goiás	Female	40 to 44	10.835873	0.8113905	17.396246
Goiás	Female	45 to 49	10.540693	0.8407297	16.544937
Goiás	Female	50 to 54	9.7290233	0.7817885	15.326303
Goiás	Female	55 to 59	8.6783021	0.7628918	13.964224
Goiás	Female	60 to 64	7.8220554	0.7013326	12.117856
Goiás	Female	65 to 69	7.3359742	0.7169573	11.812173
Goiás	Female	70 to 74	6.7542013	0.6375513	10.46484
Goiás	Female	75 to 79	6.371849	0.6497359	9.9604353
Goiás	Female	80 to 84	6.4517056	0.6982491	10.198568
Goiás	Female	85 to 89	6.4894879	0.6216024	10.23375

Goiás	Female	90 to 94	6.509932	0.6443328	10.253252
Goiás	Female	95 plus	6.485663	0.6148738	10.175351
Goiás	Male	25 to 29	12.457993	0.7634306	19.850515
Goiás	Male	30 to 34	12.55887	0.8066203	20.228275
Goiás	Male	35 to 39	12.658864	0.8277954	19.847858
Goiás	Male	40 to 44	10.783318	0.7598989	16.887095
Goiás	Male	45 to 49	10.375187	0.7065096	16.656194
Goiás	Male	50 to 54	9.8210206	0.6328683	15.808782
Goiás	Male	55 to 59	8.6205377	0.6463051	13.845525
Goiás	Male	60 to 64	7.7521281	0.5420343	12.028966
Goiás	Male	65 to 69	7.1485689	0.5716087	11.232975
Goiás	Male	70 to 74	6.7455856	0.5431724	10.534408
Goiás	Male	75 to 79	6.2686993	0.5280564	9.9105748
Goiás	Male	80 to 84	6.4908953	0.558765	10.464635
Goiás	Male	85 to 89	6.434481	0.5974722	10.199114
Goiás	Male	90 to 94	6.4059705	0.5791415	10.072272
Goiás	Male	95 plus	6.431114	0.5302432	10.171425
Maranhão	Female	25 to 29	12.658337	0.8771262	20.37614
Maranhão	Female	30 to 34	12.705863	1.012439	19.645661
Maranhão	Female	35 to 39	12.819245	0.9991118	19.842337
Maranhão	Female	40 to 44	10.85436	0.7979081	17.292606
Maranhão	Female	45 to 49	10.595832	0.8785918	16.650082
Maranhão	Female	50 to 54	9.7713681	0.794425	15.435155
Maranhão	Female	55 to 59	8.688001	0.7672297	13.891922
Maranhão	Female	60 to 64	7.8327167	0.6757784	12.279179
Maranhão	Female	65 to 69	7.3102454	0.6574801	11.567432
Maranhão	Female	70 to 74	6.7268111	0.6857452	10.816598
Maranhão	Female	75 to 79	6.4426516	0.6250099	10.190719
Maranhão	Female	80 to 84	6.4467512	0.7241328	9.9995246
Maranhão	Female	85 to 89	6.5301701	0.6073805	10.284784
Maranhão	Female	90 to 94	6.5039681	0.5826773	9.8076234
Maranhão	Female	95 plus	6.4798571	0.5892164	9.9485245
Maranhão	Male	25 to 29	12.440218	0.8338819	19.250014
Maranhão	Male	30 to 34	12.546033	0.8431153	20.056777
Maranhão	Male	35 to 39	12.646873	0.7991227	20.201234
Maranhão	Male	40 to 44	10.742019	0.7220101	17.058282
Maranhão	Male	45 to 49	10.387951	0.7156878	16.452557
Maranhão	Male	50 to 54	9.7364007	0.6947371	15.804935
Maranhão	Male	55 to 59	8.6267875	0.634741	13.77801
Maranhão	Male	60 to 64	7.681429	0.5908287	11.907447
Maranhão	Male	65 to 69	7.2072037	0.5788183	11.530829
Maranhão	Male	70 to 74	6.695734	0.618425	10.575204
Maranhão	Male	75 to 79	6.2517172	0.5273301	9.7493991

Maranhão	Male	80 to 84	6.4849621	0.5392971	10.320706
Maranhão	Male	85 to 89	6.4295782	0.5459379	10.305972
Maranhão	Male	90 to 94	6.416773	0.5470598	10.224608
Maranhão	Male	95 plus	6.3872195	0.5193308	9.9474976
Mato Grosso	Female	25 to 29	12.70446	0.9504772	20.503911
Mato Grosso	Female	30 to 34	12.667225	1.008382	19.913301
Mato Grosso	Female	35 to 39	12.803003	0.9981091	20.165073
Mato Grosso	Female	40 to 44	10.815945	0.8651154	17.019852
Mato Grosso	Female	45 to 49	10.57055	0.9040883	16.168689
Mato Grosso	Female	50 to 54	9.850647	0.8380412	15.039618
Mato Grosso	Female	55 to 59	8.715805	0.7408574	13.541403
Mato Grosso	Female	60 to 64	7.8154342	0.7822378	12.179165
Mato Grosso	Female	65 to 69	7.3072992	0.7046569	11.306731
Mato Grosso	Female	70 to 74	6.7377616	0.6384864	10.632077
Mato Grosso	Female	75 to 79	6.3800217	0.6954678	9.616012
Mato Grosso	Female	80 to 84	6.5186396	0.6210563	10.156576
Mato Grosso	Female	85 to 89	6.4915735	0.6586925	10.376387
Mato Grosso	Female	90 to 94	6.5147687	0.5885376	10.108953
Mato Grosso	Female	95 plus	6.4582577	0.6167726	9.8491578
Mato Grosso	Male	25 to 29	12.509942	0.8281974	19.986777
Mato Grosso	Male	30 to 34	12.504616	0.8268796	20.192063
Mato Grosso	Male	35 to 39	12.599233	0.7560064	19.862356
Mato Grosso	Male	40 to 44	10.760351	0.7160605	17.389452
Mato Grosso	Male	45 to 49	10.448904	0.7173709	17.11429
Mato Grosso	Male	50 to 54	9.7879514	0.6825547	15.615571
Mato Grosso	Male	55 to 59	8.6590973	0.5951884	13.74264
Mato Grosso	Male	60 to 64	7.7510719	0.5536934	12.276695
Mato Grosso	Male	65 to 69	7.1795457	0.5929347	11.475533
Mato Grosso	Male	70 to 74	6.6833745	0.5536367	10.845664
Mato Grosso	Male	75 to 79	6.2722142	0.5070792	9.9201831
Mato Grosso	Male	80 to 84	6.4740417	0.5913264	10.423935
Mato Grosso	Male	85 to 89	6.4562843	0.5871415	10.328664
Mato Grosso	Male	90 to 94	6.3790921	0.6227541	10.020765
Mato Grosso	Male	95 plus	6.4427167	0.5469633	10.392679
Mato Grosso do Sul	Female	25 to 29	12.671565	0.8047126	19.96418
Mato Grosso do Sul	Female	30 to 34	12.694248	0.8802826	19.599199
Mato Grosso do Sul	Female	35 to 39	12.845268	1.0282079	20.066695
Mato Grosso do Sul	Female	40 to 44	10.886957	0.8308264	17.538352
Mato Grosso do Sul	Female	45 to 49	10.622757	0.8822643	16.193036
Mato Grosso do Sul	Female	50 to 54	9.8143222	0.809411	15.560142
Mato Grosso do Sul	Female	55 to 59	8.6914958	0.7628924	13.760091
Mato Grosso do Sul	Female	60 to 64	7.8451773	0.7290951	12.384051
Mato Grosso do Sul	Female	65 to 69	7.3088719	0.7187728	11.309009

Mato Grosso do Sul	Female	70 to 74	6.7475143	0.6323226	10.612138
Mato Grosso do Sul	Female	75 to 79	6.4047389	0.5729605	9.8965368
Mato Grosso do Sul	Female	80 to 84	6.4388738	0.6181806	10.19
Mato Grosso do Sul	Female	85 to 89	6.5318417	0.6110754	10.142426
Mato Grosso do Sul	Female	90 to 94	6.4984909	0.6258687	10.035934
Mato Grosso do Sul	Female	95 plus	6.4601031	0.6363447	10.125872
Mato Grosso do Sul	Male	25 to 29	12.410052	0.7957066	19.770456
Mato Grosso do Sul	Male	30 to 34	12.528912	0.7520525	20.070002
Mato Grosso do Sul	Male	35 to 39	12.77042	0.9237343	20.02696
Mato Grosso do Sul	Male	40 to 44	10.803788	0.728902	17.548604
Mato Grosso do Sul	Male	45 to 49	10.377778	0.7022614	16.660419
Mato Grosso do Sul	Male	50 to 54	9.8329311	0.6891646	15.929671
Mato Grosso do Sul	Male	55 to 59	8.6387875	0.6523634	13.948083
Mato Grosso do Sul	Male	60 to 64	7.712397	0.6013165	12.121437
Mato Grosso do Sul	Male	65 to 69	7.214761	0.6044342	11.490454
Mato Grosso do Sul	Male	70 to 74	6.7145137	0.5193184	10.714842
Mato Grosso do Sul	Male	75 to 79	6.3090546	0.5119457	9.8559734
Mato Grosso do Sul	Male	80 to 84	6.4747995	0.5392248	10.469706
Mato Grosso do Sul	Male	85 to 89	6.4598918	0.5770845	10.396855
Mato Grosso do Sul	Male	90 to 94	6.4301346	0.6135591	10.542553
Mato Grosso do Sul	Male	95 plus	6.4307464	0.5538944	10.338453
Minas Gerais	Female	25 to 29	12.633386	0.9185713	19.50815
Minas Gerais	Female	30 to 34	12.661884	0.8911772	19.778699
Minas Gerais	Female	35 to 39	12.790693	1.0283626	19.705634
Minas Gerais	Female	40 to 44	10.768779	0.8336691	16.54843
Minas Gerais	Female	45 to 49	10.460623	0.8980273	15.930024
Minas Gerais	Female	50 to 54	9.837451	0.7661889	15.321848
Minas Gerais	Female	55 to 59	8.7116501	0.8014634	13.815509
Minas Gerais	Female	60 to 64	7.8314101	0.7105962	12.264397
Minas Gerais	Female	65 to 69	7.2812601	0.696377	11.319186
Minas Gerais	Female	70 to 74	6.7436712	0.6908472	10.516027
Minas Gerais	Female	75 to 79	6.3717868	0.5563227	9.9422495
Minas Gerais	Female	80 to 84	6.4116178	0.7318883	9.9227627
Minas Gerais	Female	85 to 89	6.4813249	0.5658617	9.8676082
Minas Gerais	Female	90 to 94	6.4744682	0.6234984	10.028903
Minas Gerais	Female	95 plus	6.432223	0.6961659	9.7408711
Minas Gerais	Male	25 to 29	12.484046	0.8740368	19.560909
Minas Gerais	Male	30 to 34	12.434921	0.8111061	19.862155
Minas Gerais	Male	35 to 39	12.582702	0.7530542	19.771481
Minas Gerais	Male	40 to 44	10.75445	0.7259128	16.797279
Minas Gerais	Male	45 to 49	10.449513	0.690697	16.72245
Minas Gerais	Male	50 to 54	9.7745427	0.6665036	15.733172
Minas Gerais	Male	55 to 59	8.6157381	0.6220544	13.439072

Minas Gerais	Male	60 to 64	7.7262866	0.5636497	12.106308
Minas Gerais	Male	65 to 69	7.1749979	0.6412762	11.398845
Minas Gerais	Male	70 to 74	6.7149715	0.5474817	10.369052
Minas Gerais	Male	75 to 79	6.2845731	0.5087695	9.7521912
Minas Gerais	Male	80 to 84	6.4554884	0.5402205	10.063915
Minas Gerais	Male	85 to 89	6.4226231	0.5413698	9.9484189
Minas Gerais	Male	90 to 94	6.364563	0.5325158	9.9880025
Minas Gerais	Male	95 plus	6.3740138	0.5385061	9.9140953
Pará	Female	25 to 29	12.686303	0.9253243	19.801994
Pará	Female	30 to 34	12.718449	0.9840484	19.663042
Pará	Female	35 to 39	12.7853	0.949949	20.20093
Pará	Female	40 to 44	10.79301	0.8265282	16.908413
Pará	Female	45 to 49	10.579663	0.8359706	16.542805
Pará	Female	50 to 54	9.8322889	0.7567324	15.409908
Pará	Female	55 to 59	8.6971287	0.7650945	13.657844
Pará	Female	60 to 64	7.7798965	0.7263103	12.039035
Pará	Female	65 to 69	7.2741163	0.606398	11.391633
Pará	Female	70 to 74	6.7433975	0.6421853	10.793675
Pará	Female	75 to 79	6.4185262	0.6258375	9.9703274
Pará	Female	80 to 84	6.4250478	0.742241	10.075325
Pará	Female	85 to 89	6.4932553	0.6303518	10.021368
Pará	Female	90 to 94	6.5018835	0.593421	9.9280501
Pará	Female	95 plus	6.4705276	0.6053626	10.026678
Pará	Male	25 to 29	12.352107	0.7153055	19.258179
Pará	Male	30 to 34	12.505317	0.8623871	19.958304
Pará	Male	35 to 39	12.685549	0.842892	20.311244
Pará	Male	40 to 44	10.691342	0.708832	16.956692
Pará	Male	45 to 49	10.446756	0.7177354	16.461
Pará	Male	50 to 54	9.7725443	0.6919383	15.660215
Pará	Male	55 to 59	8.6427681	0.628814	13.487493
Pará	Male	60 to 64	7.697825	0.6519036	12.07901
Pará	Male	65 to 69	7.2459634	0.6089317	11.38531
Pará	Male	70 to 74	6.6734322	0.5237466	10.651605
Pará	Male	75 to 79	6.2713312	0.5354191	9.7139638
Pará	Male	80 to 84	6.4524253	0.5276581	10.214737
Pará	Male	85 to 89	6.4357434	0.569236	10.277268
Pará	Male	90 to 94	6.4143266	0.594208	10.215383
Pará	Male	95 plus	6.391851	0.5571621	10.36576
Paraíba	Female	25 to 29	12.745736	0.8844606	19.991086
Paraíba	Female	30 to 34	12.736931	0.8553715	19.864632
Paraíba	Female	35 to 39	12.760039	0.986809	19.800397
Paraíba	Female	40 to 44	10.897318	0.9296698	17.509209
Paraíba	Female	45 to 49	10.567911	0.8956039	16.74712

Paraíba	Female	50 to 54	9.7836759	0.8008278	15.30678
Paraíba	Female	55 to 59	8.6845549	0.7699661	13.623356
Paraíba	Female	60 to 64	7.8488995	0.7160491	11.960844
Paraíba	Female	65 to 69	7.3317353	0.6502492	11.701716
Paraíba	Female	70 to 74	6.7170279	0.7034313	10.787805
Paraíba	Female	75 to 79	6.3999127	0.6271879	10.246377
Paraíba	Female	80 to 84	6.4465733	0.6701913	10.224382
Paraíba	Female	85 to 89	6.4927816	0.6320938	10.333759
Paraíba	Female	90 to 94	6.5704663	0.6349999	10.475172
Paraíba	Female	95 plus	6.4919614	0.5918068	10.426161
Paraíba	Male	25 to 29	12.497425	0.8024507	20.093103
Paraíba	Male	30 to 34	12.564299	0.8089684	19.863457
Paraíba	Male	35 to 39	12.601354	0.7546975	19.928108
Paraíba	Male	40 to 44	10.855769	0.7383105	17.486549
Paraíba	Male	45 to 49	10.423152	0.7135635	16.771085
Paraíba	Male	50 to 54	9.7734355	0.685141	15.505521
Paraíba	Male	55 to 59	8.6578744	0.6245162	13.418776
Paraíba	Male	60 to 64	7.7369907	0.6479195	12.093215
Paraíba	Male	65 to 69	7.2077371	0.6204736	11.451734
Paraíba	Male	70 to 74	6.7306328	0.558801	10.862506
Paraíba	Male	75 to 79	6.295536	0.5449127	9.9930816
Paraíba	Male	80 to 84	6.5169458	0.5909691	10.43603
Paraíba	Male	85 to 89	6.4306156	0.5712966	10.320474
Paraíba	Male	90 to 94	6.420991	0.5773317	10.282183
Paraíba	Male	95 plus	6.4011602	0.5455501	10.28489
Paraná	Female	25 to 29	12.676644	0.8810284	19.884367
Paraná	Female	30 to 34	12.686786	1.009372	19.831271
Paraná	Female	35 to 39	12.923676	1.0151874	20.226566
Paraná	Female	40 to 44	10.749689	0.8438411	16.978365
Paraná	Female	45 to 49	10.551694	0.9037705	16.319176
Paraná	Female	50 to 54	9.8127771	0.8151948	15.102527
Paraná	Female	55 to 59	8.7099527	0.7781602	13.922924
Paraná	Female	60 to 64	7.792004	0.6636687	12.12429
Paraná	Female	65 to 69	7.2996692	0.6525706	11.473093
Paraná	Female	70 to 74	6.6547296	0.6406566	10.719944
Paraná	Female	75 to 79	6.4272592	0.5851473	10.032237
Paraná	Female	80 to 84	6.4475586	0.667477	10.087941
Paraná	Female	85 to 89	6.4487519	0.5722578	10.148413
Paraná	Female	90 to 94	6.5185812	0.5612361	10.200608
Paraná	Female	95 plus	6.4727953	0.6341604	10.101367
Paraná	Male	25 to 29	12.456625	0.8203828	19.60798
Paraná	Male	30 to 34	12.533981	0.7656091	19.937531
Paraná	Male	35 to 39	12.702334	0.8826657	19.826232

Paraná	Male	40 to 44	10.797002	0.8017563	17.132139
Paraná	Male	45 to 49	10.349721	0.7201757	16.227636
Paraná	Male	50 to 54	9.8067971	0.6958309	15.364303
Paraná	Male	55 to 59	8.735667	0.7099133	13.811544
Paraná	Male	60 to 64	7.7093672	0.6158553	11.891751
Paraná	Male	65 to 69	7.1817786	0.5661808	11.171451
Paraná	Male	70 to 74	6.6837127	0.5685868	10.599544
Paraná	Male	75 to 79	6.2380925	0.528799	9.8362921
Paraná	Male	80 to 84	6.4503517	0.5952302	10.16205
Paraná	Male	85 to 89	6.4647062	0.5574131	10.433265
Paraná	Male	90 to 94	6.3702986	0.5507119	9.9762432
Paraná	Male	95 plus	6.4085582	0.5693325	10.333152
Pernambuco	Female	25 to 29	12.629725	0.8971207	19.844049
Pernambuco	Female	30 to 34	12.679704	0.9382871	20.054539
Pernambuco	Female	35 to 39	12.801214	0.9765486	19.633373
Pernambuco	Female	40 to 44	10.899951	0.8741626	17.373277
Pernambuco	Female	45 to 49	10.566773	0.8868696	16.333697
Pernambuco	Female	50 to 54	9.8192614	0.8694031	15.168365
Pernambuco	Female	55 to 59	8.6889648	0.766617	13.635069
Pernambuco	Female	60 to 64	7.7834863	0.7341544	11.933938
Pernambuco	Female	65 to 69	7.3073264	0.6717054	11.584964
Pernambuco	Female	70 to 74	6.7625457	0.6436626	10.767012
Pernambuco	Female	75 to 79	6.3533739	0.6300454	9.9514911
Pernambuco	Female	80 to 84	6.4530618	0.6511871	10.008159
Pernambuco	Female	85 to 89	6.4944614	0.6251038	10.194513
Pernambuco	Female	90 to 94	6.5078774	0.6311094	10.194615
Pernambuco	Female	95 plus	6.4571078	0.6064166	10.11829
Pernambuco	Male	25 to 29	12.439098	0.8005783	19.131251
Pernambuco	Male	30 to 34	12.523954	0.8257716	20.072304
Pernambuco	Male	35 to 39	12.643493	0.8241895	20.07771
Pernambuco	Male	40 to 44	10.709044	0.733544	17.12622
Pernambuco	Male	45 to 49	10.372585	0.67784	16.348062
Pernambuco	Male	50 to 54	9.7879877	0.6702107	15.690418
Pernambuco	Male	55 to 59	8.6943112	0.6505999	13.776119
Pernambuco	Male	60 to 64	7.7193173	0.5651532	12.057461
Pernambuco	Male	65 to 69	7.184777	0.5924983	11.426672
Pernambuco	Male	70 to 74	6.6786273	0.5854705	10.587912
Pernambuco	Male	75 to 79	6.2802121	0.5190576	9.7468729
Pernambuco	Male	80 to 84	6.4807147	0.5332967	10.348756
Pernambuco	Male	85 to 89	6.4303044	0.6018029	10.292806
Pernambuco	Male	90 to 94	6.4130184	0.5652299	10.307873
Pernambuco	Male	95 plus	6.3962976	0.5559382	10.191848
Piauí	Female	25 to 29	12.621643	0.989328	19.14009

Piauí	Female	30 to 34	12.697708	0.9472197	20.05259
Piauí	Female	35 to 39	12.878851	1.0048351	20.421934
Piauí	Female	40 to 44	10.869589	0.7906426	17.056015
Piauí	Female	45 to 49	10.621258	0.9054276	16.689223
Piauí	Female	50 to 54	9.7589354	0.8432688	15.156703
Piauí	Female	55 to 59	8.7349088	0.7935861	14.006192
Piauí	Female	60 to 64	7.7962423	0.7179154	12.193526
Piauí	Female	65 to 69	7.3172525	0.6795935	11.442601
Piauí	Female	70 to 74	6.7350326	0.6556466	10.707688
Piauí	Female	75 to 79	6.4208172	0.6388964	10.104116
Piauí	Female	80 to 84	6.4665657	0.6421203	9.9880802
Piauí	Female	85 to 89	6.49325	0.5585648	9.945409
Piauí	Female	90 to 94	6.4920867	0.6372249	10.01781
Piauí	Female	95 plus	6.5302939	0.6409818	10.077872
Piauí	Male	25 to 29	12.431438	0.8134466	19.458134
Piauí	Male	30 to 34	12.485168	0.8129245	19.796549
Piauí	Male	35 to 39	12.750942	0.8909942	20.099956
Piauí	Male	40 to 44	10.747912	0.6815682	17.362959
Piauí	Male	45 to 49	10.399644	0.7113261	16.541413
Piauí	Male	50 to 54	9.7663396	0.6821753	15.605546
Piauí	Male	55 to 59	8.6700416	0.6183712	13.90089
Piauí	Male	60 to 64	7.7665479	0.59102	12.582238
Piauí	Male	65 to 69	7.1904874	0.6106835	11.019724
Piauí	Male	70 to 74	6.7499333	0.521357	10.955676
Piauí	Male	75 to 79	6.2665334	0.5325538	9.8137954
Piauí	Male	80 to 84	6.4530452	0.5258017	10.19204
Piauí	Male	85 to 89	6.3865976	0.5406208	10.154036
Piauí	Male	90 to 94	6.3823986	0.578183	10.124888
Piauí	Male	95 plus	6.3744965	0.5997772	10.303124
Rio de Janeiro	Female	25 to 29	12.635594	0.9619468	19.671785
Rio de Janeiro	Female	30 to 34	12.747587	0.9800306	20.009672
Rio de Janeiro	Female	35 to 39	12.858127	0.9783665	20.115892
Rio de Janeiro	Female	40 to 44	10.847695	0.8404222	16.928074
Rio de Janeiro	Female	45 to 49	10.516029	0.9227071	16.229518
Rio de Janeiro	Female	50 to 54	9.7789003	0.830553	15.270339
Rio de Janeiro	Female	55 to 59	8.6824431	0.8197354	13.724661
Rio de Janeiro	Female	60 to 64	7.8717822	0.7261597	12.264186
Rio de Janeiro	Female	65 to 69	7.2771149	0.7227834	11.139901
Rio de Janeiro	Female	70 to 74	6.7314772	0.6232086	10.437474
Rio de Janeiro	Female	75 to 79	6.4356501	0.6088737	9.9823831
Rio de Janeiro	Female	80 to 84	6.46456	0.6769143	10.115098
Rio de Janeiro	Female	85 to 89	6.4764901	0.5983682	9.9174516
Rio de Janeiro	Female	90 to 94	6.5073586	0.5745856	9.7142317

Rio de Janeiro	Female	95 plus	6.4597108	0.6129647	9.7666372
Rio de Janeiro	Male	25 to 29	12.505333	0.8026924	19.532642
Rio de Janeiro	Male	30 to 34	12.525852	0.8508608	20.235305
Rio de Janeiro	Male	35 to 39	12.697094	0.8226511	19.944831
Rio de Janeiro	Male	40 to 44	10.694525	0.6632062	17.031594
Rio de Janeiro	Male	45 to 49	10.330401	0.7011723	16.541154
Rio de Janeiro	Male	50 to 54	9.8067594	0.6280075	15.297894
Rio de Janeiro	Male	55 to 59	8.6347575	0.6228168	13.557886
Rio de Janeiro	Male	60 to 64	7.7155149	0.5814937	12.117856
Rio de Janeiro	Male	65 to 69	7.1714257	0.6231343	11.138884
Rio de Janeiro	Male	70 to 74	6.6795755	0.5238624	10.606722
Rio de Janeiro	Male	75 to 79	6.2457754	0.5421067	9.6684783
Rio de Janeiro	Male	80 to 84	6.43112	0.5403076	9.8675506
Rio de Janeiro	Male	85 to 89	6.3934328	0.5741069	10.125544
Rio de Janeiro	Male	90 to 94	6.3807048	0.5835686	9.8983684
Rio de Janeiro	Male	95 plus	6.4071997	0.5511042	10.059257
Rio Grande do Norte	Female	25 to 29	12.732177	0.9292878	19.690596
Rio Grande do Norte	Female	30 to 34	12.671999	0.9838355	19.650582
Rio Grande do Norte	Female	35 to 39	12.872069	1.0584508	20.480616
Rio Grande do Norte	Female	40 to 44	10.773889	0.8213211	16.863871
Rio Grande do Norte	Female	45 to 49	10.543673	0.8830395	16.7254
Rio Grande do Norte	Female	50 to 54	9.8638002	0.8583145	15.349984
Rio Grande do Norte	Female	55 to 59	8.6822243	0.7541129	13.763335
Rio Grande do Norte	Female	60 to 64	7.864161	0.7306763	12.434684
Rio Grande do Norte	Female	65 to 69	7.3109988	0.7205285	11.576693
Rio Grande do Norte	Female	70 to 74	6.7285661	0.6349219	10.32782
Rio Grande do Norte	Female	75 to 79	6.436127	0.635724	10.028742
Rio Grande do Norte	Female	80 to 84	6.4564549	0.6524782	10.013896
Rio Grande do Norte	Female	85 to 89	6.536778	0.5583378	10.163808
Rio Grande do Norte	Female	90 to 94	6.4861759	0.6002357	10.209239
Rio Grande do Norte	Female	95 plus	6.4685732	0.6318155	10.08144
Rio Grande do Norte	Male	25 to 29	12.449973	0.7543214	19.548723
Rio Grande do Norte	Male	30 to 34	12.438778	0.7933613	19.877168
Rio Grande do Norte	Male	35 to 39	12.645895	0.7656354	20.227317
Rio Grande do Norte	Male	40 to 44	10.749111	0.7028172	17.066447
Rio Grande do Norte	Male	45 to 49	10.419701	0.7155293	16.630486
Rio Grande do Norte	Male	50 to 54	9.80233	0.6733996	15.978689
Rio Grande do Norte	Male	55 to 59	8.6432133	0.6218216	13.841415
Rio Grande do Norte	Male	60 to 64	7.7102087	0.5887597	12.231353
Rio Grande do Norte	Male	65 to 69	7.2044126	0.5680637	11.269461
Rio Grande do Norte	Male	70 to 74	6.7137551	0.5639791	10.598938
Rio Grande do Norte	Male	75 to 79	6.2575283	0.5354354	9.8321596
Rio Grande do Norte	Male	80 to 84	6.45402	0.5355983	10.529875

Rio Grande do Norte	Male	85 to 89	6.4628111	0.5426417	10.532273
Rio Grande do Norte	Male	90 to 94	6.4052168	0.5334142	9.9876373
Rio Grande do Norte	Male	95 plus	6.4154447	0.5509895	10.113376
Rio Grande do Sul	Female	25 to 29	12.6382	1.0617337	19.612407
Rio Grande do Sul	Female	30 to 34	12.697849	1.0353963	19.924062
Rio Grande do Sul	Female	35 to 39	12.838195	1.0566566	19.943867
Rio Grande do Sul	Female	40 to 44	10.848219	0.7821944	17.408622
Rio Grande do Sul	Female	45 to 49	10.556359	0.927035	16.240681
Rio Grande do Sul	Female	50 to 54	9.8235325	0.7519135	15.581694
Rio Grande do Sul	Female	55 to 59	8.7084788	0.8087863	13.748381
Rio Grande do Sul	Female	60 to 64	7.8184919	0.7283322	12.212279
Rio Grande do Sul	Female	65 to 69	7.2590494	0.7206845	11.228789
Rio Grande do Sul	Female	70 to 74	6.7678312	0.6442007	10.584871
Rio Grande do Sul	Female	75 to 79	6.4029247	0.6687445	9.8081509
Rio Grande do Sul	Female	80 to 84	6.4752176	0.5890998	10.12333
Rio Grande do Sul	Female	85 to 89	6.5241124	0.630655	10.115457
Rio Grande do Sul	Female	90 to 94	6.5048863	0.6370866	10.204272
Rio Grande do Sul	Female	95 plus	6.4915224	0.58855	10.083388
Rio Grande do Sul	Male	25 to 29	12.575973	0.8927678	19.999708
Rio Grande do Sul	Male	30 to 34	12.575694	0.8213654	20.523536
Rio Grande do Sul	Male	35 to 39	12.665597	0.8169243	19.632498
Rio Grande do Sul	Male	40 to 44	10.756336	0.7182139	17.369882
Rio Grande do Sul	Male	45 to 49	10.409699	0.7044961	16.471224
Rio Grande do Sul	Male	50 to 54	9.7625154	0.6911895	15.534153
Rio Grande do Sul	Male	55 to 59	8.6588267	0.5915701	13.831619
Rio Grande do Sul	Male	60 to 64	7.7337395	0.5758061	12.255835
Rio Grande do Sul	Male	65 to 69	7.1614546	0.5785295	11.15445
Rio Grande do Sul	Male	70 to 74	6.7109722	0.5467143	10.495747
Rio Grande do Sul	Male	75 to 79	6.2575953	0.5841007	9.7771782
Rio Grande do Sul	Male	80 to 84	6.4864666	0.5651287	10.328299
Rio Grande do Sul	Male	85 to 89	6.4304299	0.557378	10.12092
Rio Grande do Sul	Male	90 to 94	6.4034969	0.5499232	10.190242
Rio Grande do Sul	Male	95 plus	6.4151297	0.5560752	10.416181
Rondônia	Female	25 to 29	12.665591	0.8926959	20.033012
Rondônia	Female	30 to 34	12.705839	0.8906134	19.565408
Rondônia	Female	35 to 39	12.838417	1.0261917	20.107504
Rondônia	Female	40 to 44	10.8412	0.833319	17.181387
Rondônia	Female	45 to 49	10.542902	0.8950628	16.602305
Rondônia	Female	50 to 54	9.770343	0.778552	15.308623
Rondônia	Female	55 to 59	8.6753836	0.7509673	13.301457
Rondônia	Female	60 to 64	7.8158314	0.6792501	12.193491
Rondônia	Female	65 to 69	7.2599113	0.6254345	11.292591
Rondônia	Female	70 to 74	6.7041384	0.6816415	10.617778

Rondônia	Female	75 to 79	6.4094056	0.6580882	9.9267136
Rondônia	Female	80 to 84	6.4328314	0.6672613	9.9330812
Rondônia	Female	85 to 89	6.4904042	0.5819942	10.161738
Rondônia	Female	90 to 94	6.4827939	0.6420976	10.124992
Rondônia	Female	95 plus	6.4341933	0.6520673	9.9736442
Rondônia	Male	25 to 29	12.541661	0.8078348	20.007979
Rondônia	Male	30 to 34	12.508276	0.8216541	19.712843
Rondônia	Male	35 to 39	12.675105	0.8412003	20.323252
Rondônia	Male	40 to 44	10.758985	0.7076682	17.268368
Rondônia	Male	45 to 49	10.450351	0.7209291	16.389913
Rondônia	Male	50 to 54	9.8328696	0.6692238	15.464948
Rondônia	Male	55 to 59	8.6978163	0.6703656	13.86114
Rondônia	Male	60 to 64	7.7611727	0.6896341	12.379896
Rondônia	Male	65 to 69	7.1825486	0.6353473	11.411068
Rondônia	Male	70 to 74	6.6968107	0.5369084	10.70137
Rondônia	Male	75 to 79	6.2991156	0.5417537	10.038997
Rondônia	Male	80 to 84	6.4473662	0.5607638	10.446682
Rondônia	Male	85 to 89	6.4344166	0.5622823	10.293085
Rondônia	Male	90 to 94	6.3976328	0.5958498	10.187578
Rondônia	Male	95 plus	6.4066741	0.5164686	10.278321
Roraima	Female	25 to 29	12.657874	0.9619689	19.665858
Roraima	Female	30 to 34	12.648781	0.9131886	19.470233
Roraima	Female	35 to 39	12.805243	0.9968298	19.703037
Roraima	Female	40 to 44	10.898994	0.8622164	16.866759
Roraima	Female	45 to 49	10.572464	0.9159139	16.71521
Roraima	Female	50 to 54	9.7902397	0.8769744	15.218734
Roraima	Female	55 to 59	8.7212325	0.7569711	13.714489
Roraima	Female	60 to 64	7.8343266	0.7064129	12.090329
Roraima	Female	65 to 69	7.2552296	0.635042	11.35819
Roraima	Female	70 to 74	6.7274766	0.6547938	10.407017
Roraima	Female	75 to 79	6.393753	0.6357186	9.8864601
Roraima	Female	80 to 84	6.4635682	0.6519961	10.224686
Roraima	Female	85 to 89	6.4985071	0.573567	10.569846
Roraima	Female	90 to 94	6.5142476	0.6014039	10.11438
Roraima	Female	95 plus	6.476637	0.6205017	10.361912
Roraima	Male	25 to 29	12.53359	0.8304759	19.676228
Roraima	Male	30 to 34	12.53617	0.8318709	19.933661
Roraima	Male	35 to 39	12.697632	0.7304267	20.517694
Roraima	Male	40 to 44	10.743631	0.7449126	16.906639
Roraima	Male	45 to 49	10.41867	0.7585579	16.748352
Roraima	Male	50 to 54	9.7843634	0.7208284	16.078382
Roraima	Male	55 to 59	8.6920587	0.6354525	13.574086
Roraima	Male	60 to 64	7.7467391	0.5735253	12.435387

Roraima	Male	65 to 69	7.1807414	0.6261319	12.025386
Roraima	Male	70 to 74	6.7016011	0.6025166	10.699537
Roraima	Male	75 to 79	6.2580755	0.5111577	10.027313
Roraima	Male	80 to 84	6.4882765	0.5697453	10.198215
Roraima	Male	85 to 89	6.4410296	0.5232655	10.303364
Roraima	Male	90 to 94	6.3991712	0.5849389	10.116526
Roraima	Male	95 plus	6.3874925	0.5608728	10.253021
São Paulo	Female	25 to 29	12.689238	0.8670606	19.317569
São Paulo	Female	30 to 34	12.594734	0.8804754	19.568686
São Paulo	Female	35 to 39	12.775421	1.0068274	19.267993
São Paulo	Female	40 to 44	10.737953	0.839191	16.186432
São Paulo	Female	45 to 49	10.476188	0.8826008	15.764809
São Paulo	Female	50 to 54	9.7955453	0.8352679	15.139258
São Paulo	Female	55 to 59	8.6560558	0.7701137	13.483782
São Paulo	Female	60 to 64	7.7606087	0.7262509	11.97097
São Paulo	Female	65 to 69	7.2339949	0.6686694	11.220907
São Paulo	Female	70 to 74	6.7038273	0.6960976	10.323172
São Paulo	Female	75 to 79	6.3625525	0.6353433	9.6273144
São Paulo	Female	80 to 84	6.430789	0.6658955	9.6918081
São Paulo	Female	85 to 89	6.427698	0.5921348	9.8071022
São Paulo	Female	90 to 94	6.4902301	0.586039	10.056542
São Paulo	Female	95 plus	6.5005822	0.6424497	10.11228
São Paulo	Male	25 to 29	12.398949	0.8388347	19.013598
São Paulo	Male	30 to 34	12.523683	0.8208614	19.83347
São Paulo	Male	35 to 39	12.585923	0.8125456	19.487069
São Paulo	Male	40 to 44	10.711319	0.7483172	16.533871
São Paulo	Male	45 to 49	10.428936	0.6897684	16.014613
São Paulo	Male	50 to 54	9.7128552	0.6713714	14.949882
São Paulo	Male	55 to 59	8.6749195	0.6760017	13.447716
São Paulo	Male	60 to 64	7.6836565	0.6298979	11.891532
São Paulo	Male	65 to 69	7.1542633	0.6519388	11.397539
São Paulo	Male	70 to 74	6.689722	0.5157372	10.340674
São Paulo	Male	75 to 79	6.2684824	0.4943931	9.6439758
São Paulo	Male	80 to 84	6.4588462	0.5411618	10.05089
São Paulo	Male	85 to 89	6.3793903	0.5411449	9.9209415
São Paulo	Male	90 to 94	6.3805629	0.5829129	9.7785967
São Paulo	Male	95 plus	6.4075805	0.5169164	10.081652
Santa Catarina	Female	25 to 29	12.679307	0.9329061	19.512728
Santa Catarina	Female	30 to 34	12.722909	0.8519304	20.4184
Santa Catarina	Female	35 to 39	12.816291	0.9699388	20.255383
Santa Catarina	Female	40 to 44	10.798223	0.8531772	16.687144
Santa Catarina	Female	45 to 49	10.579177	0.9204347	16.85132
Santa Catarina	Female	50 to 54	9.7922343	0.787412	15.237574

Santa Catarina	Female	55 to 59	8.7045172	0.7882947	13.871562
Santa Catarina	Female	60 to 64	7.7956363	0.7443411	12.212368
Santa Catarina	Female	65 to 69	7.2948209	0.6246602	11.549008
Santa Catarina	Female	70 to 74	6.7335147	0.6208395	10.513677
Santa Catarina	Female	75 to 79	6.4312123	0.6828277	9.9571848
Santa Catarina	Female	80 to 84	6.4311823	0.6265023	9.9908306
Santa Catarina	Female	85 to 89	6.4603184	0.5890362	9.9425446
Santa Catarina	Female	90 to 94	6.4964531	0.6122728	10.088084
Santa Catarina	Female	95 plus	6.4879626	0.6397202	10.215553
Santa Catarina	Male	25 to 29	12.456661	0.7981482	19.192658
Santa Catarina	Male	30 to 34	12.560692	0.8647209	20.488579
Santa Catarina	Male	35 to 39	12.682348	0.8150464	20.407965
Santa Catarina	Male	40 to 44	10.809857	0.7452989	17.239067
Santa Catarina	Male	45 to 49	10.353847	0.6725251	16.555998
Santa Catarina	Male	50 to 54	9.7580562	0.6772362	15.409566
Santa Catarina	Male	55 to 59	8.6552431	0.6303181	13.711029
Santa Catarina	Male	60 to 64	7.75088	0.6867878	12.433449
Santa Catarina	Male	65 to 69	7.1717439	0.6284879	11.43345
Santa Catarina	Male	70 to 74	6.6657718	0.5150371	10.588546
Santa Catarina	Male	75 to 79	6.2795864	0.5315487	9.7860086
Santa Catarina	Male	80 to 84	6.4739427	0.54156	10.367398
Santa Catarina	Male	85 to 89	6.4422351	0.5701979	10.133913
Santa Catarina	Male	90 to 94	6.4348133	0.5509924	10.139477
Santa Catarina	Male	95 plus	6.379045	0.5922042	10.311012
Sergipe	Female	25 to 29	12.687709	0.9284735	19.921145
Sergipe	Female	30 to 34	12.672666	0.975524	19.660111
Sergipe	Female	35 to 39	12.84303	0.9985741	20.148724
Sergipe	Female	40 to 44	10.8153	0.8998446	17.292207
Sergipe	Female	45 to 49	10.564391	0.9102529	16.462313
Sergipe	Female	50 to 54	9.7714663	0.8058257	15.385274
Sergipe	Female	55 to 59	8.7252172	0.7726483	13.921693
Sergipe	Female	60 to 64	7.8068652	0.7112565	12.380652
Sergipe	Female	65 to 69	7.300739	0.6361039	11.379292
Sergipe	Female	70 to 74	6.7230815	0.6891651	10.43889
Sergipe	Female	75 to 79	6.3717982	0.6364233	9.9106543
Sergipe	Female	80 to 84	6.4095601	0.6589315	9.9137028
Sergipe	Female	85 to 89	6.5145631	0.5893975	10.270151
Sergipe	Female	90 to 94	6.5376026	0.6799298	10.176152
Sergipe	Female	95 plus	6.4326286	0.6017372	10.036404
Sergipe	Male	25 to 29	12.481773	0.7560199	19.619497
Sergipe	Male	30 to 34	12.525593	0.8572242	20.046176
Sergipe	Male	35 to 39	12.652475	0.8224548	19.847251
Sergipe	Male	40 to 44	10.714914	0.7571186	16.812484

Sergipe	Male	45 to 49	10.382149	0.736106	16.487421
Sergipe	Male	50 to 54	9.773569	0.6893578	15.631295
Sergipe	Male	55 to 59	8.6126044	0.6766749	13.735851
Sergipe	Male	60 to 64	7.7354906	0.5970679	12.330448
Sergipe	Male	65 to 69	7.1706129	0.567284	11.29195
Sergipe	Male	70 to 74	6.7010527	0.5811081	10.641441
Sergipe	Male	75 to 79	6.2882326	0.5193206	9.8466212
Sergipe	Male	80 to 84	6.4966693	0.5615378	10.468298
Sergipe	Male	85 to 89	6.4052113	0.5145548	10.226343
Sergipe	Male	90 to 94	6.4399516	0.5664773	10.146822
Sergipe	Male	95 plus	6.3821653	0.5531305	10.243
Tocantins	Female	25 to 29	12.645275	0.8831132	19.453428
Tocantins	Female	30 to 34	12.686766	0.9629225	19.89794
Tocantins	Female	35 to 39	12.75193	1.0518638	20.114194
Tocantins	Female	40 to 44	10.77854	0.7941449	16.828784
Tocantins	Female	45 to 49	10.567419	0.9433578	16.428874
Tocantins	Female	50 to 54	9.8524558	0.7953154	15.285487
Tocantins	Female	55 to 59	8.6787275	0.7475436	13.796893
Tocantins	Female	60 to 64	7.8215886	0.6966678	12.475501
Tocantins	Female	65 to 69	7.2802236	0.7341058	11.584495
Tocantins	Female	70 to 74	6.7268818	0.673722	10.397647
Tocantins	Female	75 to 79	6.4346333	0.6426189	10.042485
Tocantins	Female	80 to 84	6.445458	0.6944676	10.243754
Tocantins	Female	85 to 89	6.4807589	0.6333389	10.100928
Tocantins	Female	90 to 94	6.5017511	0.6147821	10.307707
Tocantins	Female	95 plus	6.4819604	0.615439	10.160945
Tocantins	Male	25 to 29	12.441459	0.7622972	19.773763
Tocantins	Male	30 to 34	12.506082	0.8227331	20.484923
Tocantins	Male	35 to 39	12.677264	0.775229	20.445777
Tocantins	Male	40 to 44	10.740046	0.7026238	17.134899
Tocantins	Male	45 to 49	10.489469	0.6459399	16.438815
Tocantins	Male	50 to 54	9.8288447	0.6797396	15.631611
Tocantins	Male	55 to 59	8.6781888	0.6235725	13.841109
Tocantins	Male	60 to 64	7.6996022	0.6107649	12.495748
Tocantins	Male	65 to 69	7.208205	0.6094679	11.679108
Tocantins	Male	70 to 74	6.7701845	0.5590634	10.965167
Tocantins	Male	75 to 79	6.2994382	0.5424019	9.9442537
Tocantins	Male	80 to 84	6.4855246	0.59369	10.227493
Tocantins	Male	85 to 89	6.4636161	0.5931186	10.156369
Tocantins	Male	90 to 94	6.4078098	0.5420221	10.144721
Tocantins	Male	95 plus	6.3840109	0.5288496	10.131285
Brazil	Female	25 to 29	12.673513	0.9440573	18.34725
Brazil	Female	30 to 34	12.675064	0.9197614	18.453381

Brazil	Female	35 to 39	12.800732	1.0342206	18.683754
Brazil	Female	40 to 44	10.798247	0.8913069	15.896464
Brazil	Female	45 to 49	10.52849	0.9691885	15.330105
Brazil	Female	50 to 54	9.8017114	0.8137018	14.36265
Brazil	Female	55 to 59	8.6882219	0.8074237	12.897949
Brazil	Female	60 to 64	7.8083459	0.7315573	11.417685
Brazil	Female	65 to 69	7.2747278	0.7013895	10.621233
Brazil	Female	70 to 74	6.7264039	0.704103	9.9398311
Brazil	Female	75 to 79	6.395696	0.631253	9.3352172
Brazil	Female	80 to 84	6.4420799	0.6808327	9.3276805
Brazil	Female	85 to 89	6.4725645	0.6146734	9.391688
Brazil	Female	90 to 94	6.4991432	0.6503321	9.5665577
Brazil	Female	95 plus	6.4710267	0.6222454	9.4425626
Brazil	Male	25 to 29	12.444545	0.8678719	18.152503
Brazil	Male	30 to 34	12.519024	0.8574677	18.988333
Brazil	Male	35 to 39	12.643506	0.8260465	18.489104
Brazil	Male	40 to 44	10.747414	0.7528922	15.818048
Brazil	Male	45 to 49	10.402381	0.7272127	15.231646
Brazil	Male	50 to 54	9.7693784	0.703863	14.492149
Brazil	Male	55 to 59	8.6543823	0.6886232	12.696074
Brazil	Male	60 to 64	7.7141471	0.6408604	11.371345
Brazil	Male	65 to 69	7.1820613	0.6224858	10.695746
Brazil	Male	70 to 74	6.7012355	0.5650945	9.7434174
Brazil	Male	75 to 79	6.2646305	0.5365664	9.0037603
Brazil	Male	80 to 84	6.4596679	0.5790928	9.4553979
Brazil	Male	85 to 89	6.4194993	0.5985133	9.4414639
Brazil	Male	90 to 94	6.3969952	0.608114	9.4131989
Brazil	Male	95 plus	6.4016126	0.5722939	9.5369594

Table S4 Number and rates of years lived with disability, per 100,000, for ischemic heart disease attributable to trans fatty acids consumption in Brazil and states, 2019.

Region	Location	Number (95% UI)	YLD rate per 100,000 (95% UI)	Age-standardized YLD rate per 100,000 (95% UI)
	Brazil	11,165.94 (932.14–18,462.69)	5.15 (0.43–8.52)	4.73 (0.40–7.76)
North	Acre	28.87 (2.25–48.05)	3.12 (0.24–5.19)	4.58 (0.37–7.61)
	Amapá	23.84 (1.93–39.54)	2.82 (0.23–4.68)	4.46 (0.37–7.46)
	Amazonas	129.91 (10.09–217.35)	3.08 (0.24–5.15)	4.42 (0.35–7.34)
	Pará	328.07 (27.19–534.40)	3.55 (0.29–5.78)	4.66 (0.39–7.63)
	Rondônia	70.99 (5.90–117.99)	4.00 (0.33–6.64)	4.55 (0.39–7.52)
	Roraima	17.45 (1.38–29.10)	2.91 (0.23–4.86)	4.46 (0.37–7.52)
	Tocantins	62.68 (5.13–105.07)	3.82 (0.31–6.40)	4.37 (0.36–7.29)
Northeast	Alagoas	153.43 (12.56–253.99)	4.19 (0.34–6.94)	4.79 (0.40–7.86)
	Bahia	791.57 (62.84–1315.48)	4.96 (0.39–8.25)	4.89 (0.39–8.19)
	Ceará	451.38 (37.89–735.92)	4.5 (0.38–7.33)	4.52 (0.39–7.37)
	Maranhão	319.25 (25.96–527.01)	3.82 (0.31–6.30)	4.86 (0.40–8.04)
	Paraíba	225.22 (18.31–373.74)	5.14 (0.42–8.53)	4.75 (0.39–7.92)
	Pernambuco	469.57 (37.37–777.35)	4.64 (0.37–7.68)	4.67 (0.37–7.77)
	Piauí	165.90 (13.93–277.00)	4.49 (0.38–7.50)	4.41 (0.37–7.39)
	Rio Grande do Norte	185.20 (14.76–305.58)	4.95 (0.39–8.17)	4.76 (0.38–7.93)
	Sergipe	107.03 (8.63–179.82)	4.44 (0.36–7.47)	4.74 (0.39–8.01)
Central-West	Federal District	106.53 (8.57–177.68)	3.52 (0.28–5.87)	3.92 (0.33–6.44)
	Goiás	320.81 (25.61–537.81)	4.67 (0.37–7.82)	4.56 (0.37–7.61)
	Mato Grosso	152.39 (12.31–252.75)	4.23 (0.34–7.02)	4.56 (0.37–7.55)
	Mato Grosso do Sul	143.51 (11.84–238.14)	5.05 (0.42–8.38)	4.85 (0.41–8.07)
Southeast	Espírito Santo	202.20 (16.47–336.23)	5.09 (0.41–8.46)	4.61 (0.38–7.66)
	Minas Gerais	1,245.31 (104.40–2,060.28)	5.74 (0.48–9.50)	4.70 (0.39–7.78)
	Rio de Janeiro	1,045.47 (88.19–1,724.44)	5.92 (0.50–9.76)	4.66 (0.39–7.67)
	São Paulo	2,654.05 (221.07–4,399.80)	5.83 (0.49–9.67)	4.87 (0.41–8.09)
South	Paraná	640.94 (54.07–1,070.14)	5.63 (0.47–9.40)	4.79 (0.41–7.97)
	Rio Grande do Sul	736.23 (61.35–1,209.25)	6.51 (0.54–10.70)	4.74 (0.39–7.81)
	Santa Catarina	388.13 (32.03–651.41)	5.42 (0.45–9.10)	4.72 (0.39–7.85)

95% UI: 95% Uncertainty interval; YLD: years lived with disability.

Table S5 The direct cost (Int$) to the Unified Health System of mediators between trans fatty acids consumption and ischemic heart disease, LDL-c, and systolic blood pressure in Brazil by type of procedure, 2019.

**Location**	**SIA**<br>**Int$ (95% UI)**	**SIH**<br>**Int$ (95% UI)**	**Total**<br>**Int$ (95% UI)**
Brazil	397,533 (29,900–617,806)	1,636,111 (135,756–2,558,975)	2,033,644 (165,656–3,176,781)

95% UI: 95% Uncertainty interval; Int$: International dollar, Int$ 1 = US$ 1; SIA: Outpatient Information System; SIH: Hospital Information System.

Table S6 The direct cost (Int$) of ischemic heart disease attributable to the trans fatty acids consumption to the Unified Health System in Brazil by states, 2019.

Region	Location	SIA Int$ (95% UI)	SIH Int$ (95% UI)	Total Int$ (95% UI)
North	Acre	120 (10–190)	57,612 (4,527–91,394)	57,732 (4,537–91,584)
	Amapá	18,254 (1,628–28,828)	107,043 (8,548–170,716)	125,297 (10,176–199,544)
	Amazonas	44,108 (4,035–69,686)	430,646 (34,339–683,910)	474,754 (38,374–753,596)
	Pará	230,062 (21,204–361,376)	492,856 (39,810–775,553)	722,918 (61,014–1,136,929)
	Rondônia	11,247 (1,005–17,841)	185,477 (15,224–293,711)	196,724 (16,229–311,552)
	Roraima	161 (15–254)	58,054 (4,647–92,334)	58,215 (4,662–92,588)
	Tocantins	11,980 (1,099–18,948)	194,607 (15,883–310,109)	206,587 (16,982–329,057)
Northeast	Alagoas	67,020 (6,105–105,772)	404,347 (33,291–640,386)	471,367 (39,396–746,158)
	Bahia	202,948 (18,366–317,532)	2,068,649 (170,146–3,260,100)	2,271,597 (188,512–3,577,632)
	Ceará	31,253 (2,859–49,067)	1,826,136 (151,960–2,880,380)	1,857,389 (154,819–2,929,447)
	Maranhão	51,973 (4,655–81,789)	359,079 (29,377–569,142)	411,052 (34,032–650,931)
	Paraíba	30,064 (2,763–47,934)	708,631 (59,360–1,120,401)	738,695 (62,123–1,168,335)
	Pernambuco	123,991 (11,296–195,500)	1,844,026 (152,118–2,904,280)	1,968,017 (163,414–3,099,780)
	Piauí	22,720 (2,041–35,695)	333,615 (27,055–528,776)	356,335 (29,096–564,471)
	Rio Grande do Norte	302,625 (26,868–477,593)	859,730 (69,529–1,362,843)	1,162,355 (96,397–1,840,436)
	Sergipe	1,154,380 (104,256–1,819,313)	341,555 (28,177–540,220)	1,495,935 (132,433–2,359,533)
Central-West	Federal District	29,351 (2,613–46,698)	531,096 (42,188–844,256)	560,447 (44,801–890,954)
	Goiás	120,959 (11,166–191,005)	1,422,136 (114,489–2,248,238)	1,543,095 (125,654–2,439,244)
	Mato Grosso	49,117 (4,506–77,497)	553,526 (44,371–876,008)	602,643 (48,877–953,505)
	Mato Grosso do Sul	32,024 (2,877–50,693)	896,239 (72,606–1,423,351)	928,263 (75,483–1,474,044)
Southeast	Espírito Santo	88,303 (8,130–139,019)	1,210,624 (100,735–1,910,324)	1,298,927 (108,865–2,049,342)
	Minas Gerais	273,619 (24,707–424,692)	6,635,359 (539,553–10,401,660)	6,908,978 (564,260–10,826,352)
	Rio de Janeiro	83,548 (7,482–129,486)	3,110,965 (253,682–4,865,567)	3,194,513 (261,164–4,995,053)
	São Paulo	15,726 (1,416–24,243)	11,901,559 (986,994–18,417,742)	11,917,325 (988,410–18,441,985)
South	Paraná	25,165 (2,246–39,729)	7,173,762 (592,294–11,256,152)	7,198,927 (594,540–11,295,881)
	Rio Grande do Sul	299,073 (26,882–469,522)	4,307,938 (350,500–6,779,499)	4,607,011 (377,382–7,249,021)
	Santa Catarina	110,614 (9,942–173,874)	3,106,517 (254,217–4,920,981)	3,217,131 (264,159–5,094,855)

95% UI: 95% Uncertainty interval; Int$: International dollar, Int$ 1 = US$ 1; SIA: Outpatient Information System; SIH: Hospital Information System.

Table S7 Population and the direct cost (Int$) per 10,000 inhabitants of ischemic heart disease attributable to the trans fatty acids consumption to the Unified Health System in Brazil by states, 2019.

Region	Location	Total Int$ (95% UI)	Population	Rate per 10,000 Int$ (95% UI)
North	Acre	57,732 (4,537–91,584)	881,935	655 (51–1,038)
	Amapá	125,297 (10,176–199,544)	845,731	1,482 (120–2,359)
	Amazonas	474,754 (38,374–753,596)	4,144,597	1,145 (93–1,818)
	Pará	722,918 (61,014–1,136,929)	8,602,865	840 (71–1322)
	Rondônia	196,724 (16,229–311,552)	1,777,225	1,107 (91–1,753)
	Roraima	58,215 (4,662–92,588)	605,761	961 (77–1528)
	Tocantins	206,587 (16,982–329,057)	1,572,866	1,313 (108–2,092)
Northeast	Alagoas	471,367 (39,396–746,158)	3,337,357	1,412 (118–2,236)
	Bahia	2,271,597 (188,512–3,577,632)	14,873,064	1,527 (127–2,405)
	Ceará	1,857,389 (154,819–2,929,447)	9,132,078	2,034 (170–3,208)
	Maranhão	411,052 (34,032–650,931)	7,075,181	581 (48–920)
	Paraíba	738,695 (62,123–1,168,335)	4,018,127	1,838 (155–2,908)
	Pernambuco	1,968,017 (163,414–3,099,780)	9,557,071	2,059 (171–3,243)
	Piauí	356,335 (29,096–564,471)	3,273,227	1089 (89–1725)
	Rio Grande do Norte	1,162,355 (96,397–1,840,436)	3,506,853	3,315 (275–5,248)
	Sergipe	1,495,935 (132,433–2,359,533)	2,298,696	6,508 (576–10,265)
Central-West	Federal District	560,447 (44,801–890,954)	3,015,268	1,859 (149–2,955)
	Goiás	1,543,095 (125,654–2,439,244)	7,018,354	2,199 (179–3,476)
	Mato Grosso	602,643 (48,877–953,505)	3,484,466	1,730 (140–2,736)
	Mato Grosso do Sul	928,263 (75,483–1,474,044)	2,778,986	3,340 (272–5,304)
Southeast	Espírito Santo	1,298,927 (108,865–2,049,342)	4,018,650	3,232 (271–5,100)
	Minas Gerais	6,908,978 (564,260–10,826,352)	21,168,791	3,264 (267–5,114)
	Rio de Janeiro	3,194,513 (261,164–4,995,053)	17,264,943	1,850 (151–2,893)
	São Paulo	11,917,325 (988,410–18,441,985)	45,919,049	2,595 (215–4,016)
South	Paraná	7,198,927 (594,540–11,295,881)	11,433,957	6,296 (520–9,879)
	Rio Grande do Sul	4,607,011 (377,382–7,249,021)	11,377,239	4,049 (332–6,372)
	Santa Catarina	3,217,131 (264,159–5,094,855)	7,164,788	4,490 (369–7,111)

95% UI: 95% Uncertainty interval; Int$: International dollar, Int$ 1 = US$ 1.

Table S8 Socio-demographic index values from Brazil and its states in 2019.

Region	Location	Socio-demographic index (SDI) 2019	Classification*
	Brazil	0.640	Middle
North	Acre	0.562	Low-middle
	Amapá	0.641	Middle
	Amazonas	0.602	Low-middle
	Pará	0.569	Low-middle
	Rondônia	0.606	Low-middle
	Roraima	0.610	Middle
	Tocantins	0.583	Low-middle
Northeast	Alagoas	0.518	Low-middle
	Bahia	0.562	Low-middle
	Ceará	0.558	Low-middle
	Maranhão	0.444	Low
	Paraíba	0.548	Low-middle
	Pernambuco	0.571	Low-middle
	Piauí	0.509	Low-middle
	Rio Grande do Norte	0.576	Low-middle
	Sergipe	0.583	Low-middle
Central-West	Federal District	0.777	High-middle
	Goiás	0.628	Middle
	Mato Grosso	0.642	Middle
	Mato Grosso do Sul	0.639	Middle
Southeast	Espírito Santo	0.660	Middle
	Minas Gerais	0.643	Middle
	Rio de Janeiro	0.702	High-middle
	São Paulo	0.702	High-middle
South	Paraná	0.662	Middle
	Rio Grande do Sul	0.684	Middle
	Santa Catarina	0.691	Middle

*Classification of the socio-demographic index (SDI) by the Global Burden of Disease Study (GBD): high (>0.81), high-middle (0.70–0.81), middle (0.61–0.69), low-middle (0.46–0.60), and low (<0.46).

References

GBD 2019 Risk Factors Collaborators. Global burden of 87 risk factors in 204 countries and territories, 1990–2019: a systematic analysis for the Global Burden of Disease Study 2019. Lancet 2020; 396: 1223–49.

Global Burden of Disease Collaborative Network. Global Burden of Disease Study 2019 (GBD 2019) Relative Risks. Seattle, United States of America: Institute for Health Metrics and Evaluation (IHME), 2020.
